# Pre-existing cell states predict resistance to multiple treatments

**DOI:** 10.1016/j.xgen.2026.101191

**Published:** 2026-03-30

**Authors:** Dylan L. Schaff, Phoebe E. White, Christopher J. Cote, Grace E. Watterson, Kevin Z. Lin, Aria J. Fasse, Nancy R. Zhang, Sydney M. Shaffer

**Affiliations:** 1 Department of Bioengineering, School of Engineering and Applied Sciences, University of Pennsylvania, Philadelphia, PA 19146, USA; 2 Department of Chemistry, School of Arts and Sciences, University of Pennsylvania, Philadelphia, PA 19146, USA; 3 Department of Biochemistry, Cell and Molecular Biology, School of Medicine, Johns Hopkins University, Baltimore, MD 21205, USA; 4 Department of Pathology, Perelman School of Medicine, University of Pennsylvania, Philadelphia, PA 19146, USA; 5 Cellular and Molecular Biology Graduate Group, University of Pennsylvania, Philadelphia, PA 19146, USA; 6 Department of Biostatistics, School of Public Health, University of Washington, Seattle, WA 98195, USA; 7 Division of Biology and Biological Engineering, California Institute of Technology, Pasadena, CA 91125, USA; 8 Department of Statistics and Data Science, The Wharton School, University of Pennsylvania, Philadelphia, PA 19146, USA

**Keywords:** scRNA-seq, clonal tracing, melanoma, treatment resistance

## Abstract

Pre-existing differences between individual cancer cells can predict which cells will become resistant to treatment. DNA barcoding methods that track clones and their cell states during treatment have furthered this understanding, previously focusing on resistance to single treatments. Here, we performed multi-treatment, high-throughput clonal tracking and single-cell RNA sequencing to trace rare clones through resistance development across many treatments in parallel, identifying cell states associated with multi-treatment resistance. We found that clones resistant to one treatment had an increased chance of separately developing resistance to other treatments. We identified high CD44 expression in treatment-naive cells as a predictor of future multi-treatment resistance. Additionally, we found that differences in pre-treatment gene expression states can lead cells within the same treatment condition to follow divergent paths toward their ultimate resistance fate. This work provides a framework for extracting targetable gene expression states from complex resistance dynamics to eliminate multi-treatment resistance.

## Highlights

• Rare cancer clones develop resistance to multiple diverse treatments
• High CD44 expression predicts multi-treatment resistance before treatment
• Clonal tracing links pre-treatment gene states to resistance outcomes
• Pre-existing transcriptional differences drive divergent resistance fates

## Graphical abstract

**Figure:**
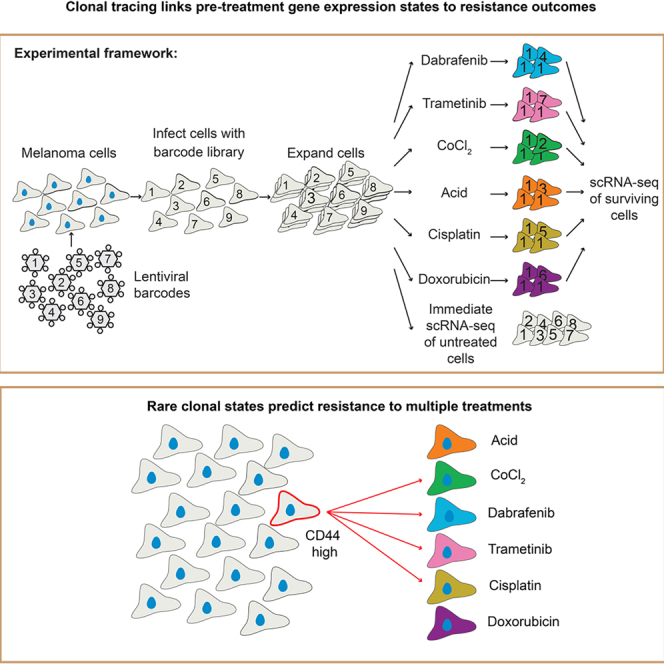

## Teaser

Schaff et al. use multi-treatment clonal tracing with single-cell RNA sequencing to show that rare melanoma clones resist multiple treatments through pre-existing gene expression states. They identify CD44 as a marker of multi-treatment resistance and show that initial transcriptional differences predict divergent resistance fates.

## Introduction

Recent years have seen remarkable progress in the development of anticancer treatments, including advances in drug delivery systems,^1^^,^^2^^,^^3^ small-molecule inhibitors,^4^^,^^5^ and immunotherapy^6^^,^^7^^,^^8^^,^^9^ that have significantly improved patient prognoses. However, cancer cells continue to evade and develop resistance to treatment.^10^^,^^11^ While resistance can be genetic in origin, with a subset of cells containing mutations that enable bypass of the drug,^12^^,^^13^^,^^14^^,^^15^ resistance can also originate from rare cells with non-genetic mechanisms of resistance, such as variability in gene expression,^16^^,^^17^^,^^18^^,^^19^^,^^20^ epigenetic state,^21^^,^^22^^,^^23^ protein levels,^24^ and cell cycle.^25^ However, studying these pre-existing states that enable resistance has been difficult, as the treatment itself dramatically alters the cellular gene expression and phenotype, thus obscuring the initial differences that enabled resistance.

To uncover these pre-existing states that enable cancer cell resistance, clonal tracing technologies that utilize DNA barcoding have emerged as powerful tools for connecting pre-treatment cellular states to post-treatment outcomes.^17^^,^^19^^,^^26^^,^^27^^,^^28^^,^^29^ These tools allow researchers to introduce barcodes into a population of cancer cells, measure gene expression in a subset of cells before treatment, expose the remaining cells to therapy, and then use the DNA barcodes to link the initial transcriptional profiles with treatment resistance. While these methods have been effective in identifying cell states that promote resistance to individual drugs, few studies to date have focused on resistance to more than a single treatment at a time.^16^^,^^17^^,^^19^^,^^27^^,^^30^^,^^31^

This focus on single treatments does not fully reflect clinical practice, where oncologists often combine or sequence multiple therapies. Unfortunately, even with such combinatorial or sequential approaches, some patients develop multi-treatment resistance.^32^^,^^33^^,^^34^^,^^35^^,^^36^^,^^37^ This resistance could arise through different models, and understanding this underlying model is important for designing more effective treatment strategies. It is possible that tumor heterogeneity might result in different subpopulations that develop resistance to specific treatments, collectively rendering the tumor resistant to multiple drugs. Alternatively, we hypothesized that there may exist rare subpopulations of cancer cells capable of adapting and developing resistance to multiple stress conditions and treatment modalities.

To test this hypothesis, we developed a multi-treatment, high-throughput barcoding framework to trace the same pool of melanoma clones over 4 weeks with a panel of treatments, including targeted inhibitors, biologically relevant selective stressors, and chemotherapeutics. Across these six treatments, we found many clones that were resistant to single treatments and, strikingly, rare clones capable of multi-treatment resistance. By combining barcoding with single-cell RNA sequencing (scRNA-seq), we uncovered gene expression states that are associated with resistance to each treatment individually and states that promote multi-treatment resistance. Single-agent resistance states contained known genes associated with resistance to BRAFi and MEKi in melanoma, including *EGFR*, *NGFR*, and *AXL*. The pre-existing population of multi-treatment-resistant cells showed high expression of *CD44* prior to treatment exposure, which we validated as a marker of cells resistant to BRAFi (dabrafenib), MEKi (trametinib), and a mimetic of hypoxic stress (CoCl_2_). Leveraging our ability to capture both the initial and resistant end states of individual clones, we demonstrated that pre-treatment gene expression profiles predict the ultimate resistance trajectories across multiple conditions, thereby elucidating the complex relationship between initial transcriptional states and diverse paths to treatment resistance.

## Results

### Rare melanoma cells develop resistance to many different treatments

To study how melanoma cells develop resistance to diverse treatments, we selected six agents with distinct mechanisms of action: two targeted inhibitors used clinically, dabrafenib^38^ (V600E mutant BRAF inhibitor) and trametinib^39^ (MEK inhibitor); two agents simulating *in vivo* selective pressures, CoCl_2_^40^^,^^41^ (mimicking hypoxia) and acidic media^42^ (simulating extracellular acidosis); and two chemotherapeutics, cisplatin^43^ (causing DNA cross-linking and breaks) and doxorubicin^44^ (inhibiting topoisomerases).

**Figure 1:**
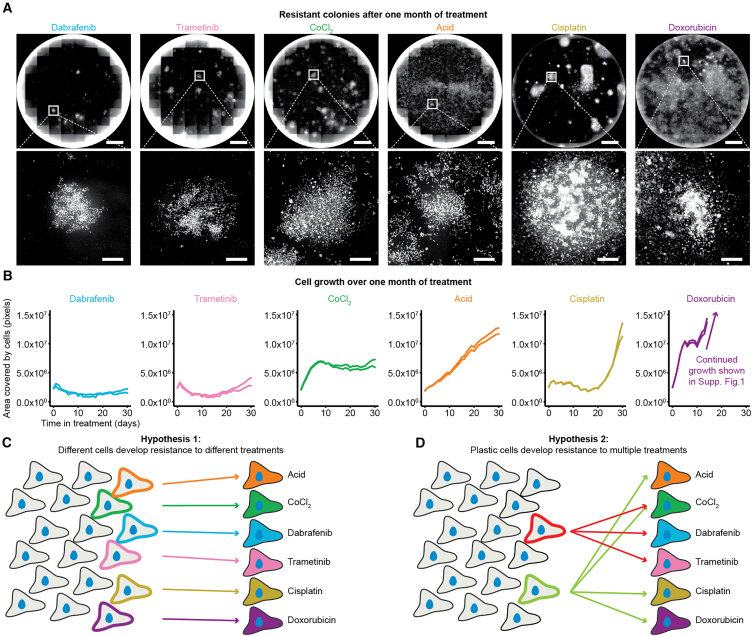
Melanoma cells develop resistance to multiple different treatments (A) (Top) Whole-well scans of WM989 A6-G3 EGFP-6xMYCNLS cells after 1 month of treatment with dabrafenib, trametinib, CoCl_2_, acidic media, cisplatin, or doxorubicin. (Bottom) Cropped images highlight individual resistant clones in each treatment. Scale bars for whole-well scans represent 5 mm, and scale bars for cropped clones represent 5 μm. (B) Area covered by cells (in pixels) daily over the course of 1 month of treatment. Doxorubicin-treated cell growth vastly outpaced other treatment conditions, so the full growth curve is plotted in Figure S1. (C) Initial hypothesis that cells with different gene expression states develop resistance to unique treatments. (D) Alternative hypothesis that cells with special gene expression states can develop resistance to multiple different treatments. See also Figure S1.

To study the frequency and rate at which resistance develops to each condition, we treated WM989 BRAF V600E melanoma cells expressing nuclear-localized EGFP with each agent individually for 1 month, imaging daily. Melanoma cells developed resistant colonies under all six treatments (Figure 1A). However, the number of resistant cells and the growth dynamics varied between conditions (Figures 1B and S1). Except for acid-treated cells, which showed monotonic growth, all conditions exhibited an initial increase in cell number followed by a decrease, with the timing and degree of cell growth before large-scale cell death varying greatly. During the period of cell death, rare cells survived and proliferated, leading to a final phase where colonies expanded. The emergence of rare, resistant cells across all treatments led us to investigate a key question: what transcriptional cell states enable only a subset of cells to grow in conditions that kill the majority of the population?

Our group and others have previously shown that cancer cells can develop resistance to single treatments through non-genetic mechanisms where differences in gene expression can predict which cells will become resistant to treatment^16^^,^^17^^,^^18^^,^^19^^,^^30^^,^^45^^,^^46^^,^^47^ (Figure 1C). However, we wondered whether cells could develop resistance to multiple treatments simultaneously. We hypothesized that this multi-treatment resistance might arise from highly plastic gene expression states that allow cells to adapt to various conditions (Figure 1D).

### Multi-treatment, high-throughput clonal tracing reveals that clones can develop resistance to unique or overlapping treatments

**Figure 2:**
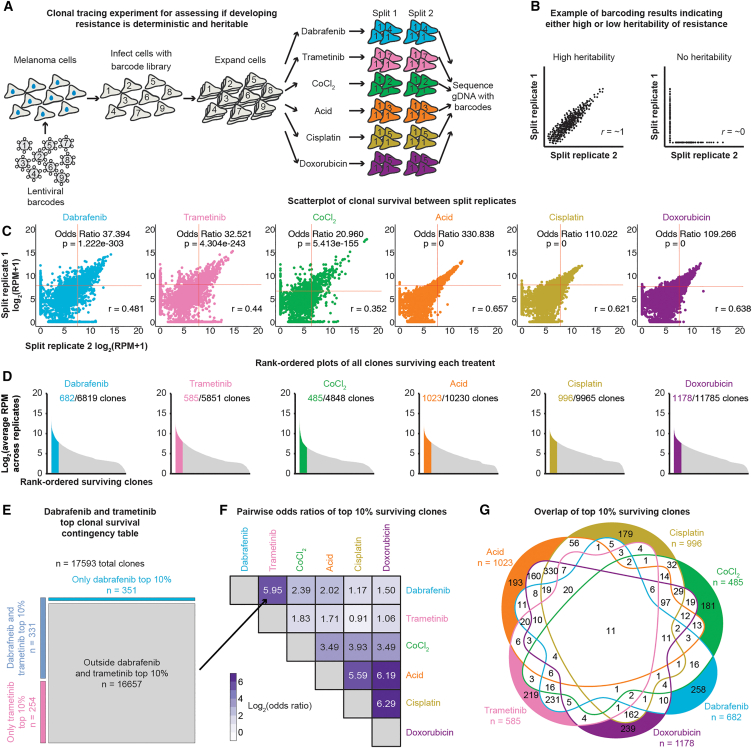
Multi-treatment, high-throughput clonal tracing reveals that clones can develop resistance to unique or overlapping treatments (A) Experimental protocol for performing multi-treatment, high-throughput clonal tracing across treatment with dabrafenib, trametinib, CoCl_2_, acidic media, cisplatin, and doxorubicin. Each different treatment was performed in duplicate (12 total splits from the expanded population) to assess whether the ability of a clone to develop resistance was conserved over six doublings. (B) Schematized examples of conditions where individual clones had similar (left, high heritability) or variable (right, low heritability) resistance to the same treatment across replicates. (C) Scatterplots of reads per million (RPM)-normalized clone abundance between replicates for each treatment (clones with an average RPM across replicates of ≤1 are excluded). Red lines mark the 90th percentile threshold. Displayed are Pearson correlations (*r* values) and odds ratios, testing whether the top 10% resistant clones in one replicate are also the top 10% in the other replicate. *p* values were calculated with Fisher’s exact test. (D) Rank-ordered bar plots of resistant clone abundance across replicates to each treatment individually. Highlighted are the top 10% most abundant of all resistant clones to each treatment. (E) Mosaic plot displaying the contingency table for calculating the odds ratio of being a top 10% resistant clone between dabrafenib and trametinib across 17,593 clones. (F) Log_2_ of pairwise odds ratios of being a top 10% resistant clone in each comparison of treatment conditions. (G) Venn diagram of top 10% resistant clones of each condition across all treatments. Filled segments of the Venn diagram highlight clones that were uniquely resistant to the given treatment.

Barcoding experiments such as ours are predicated on the assumption that cells from the same clone are essentially “copies” of each other. However, as cells divide, differences may emerge within a clone, potentially affecting their response to treatment. To test whether a clone’s resistant “fate” is predetermined and persists over cell divisions prior to treatment,^26^^,^^45^ we introduced a lentiviral library containing semi-random barcodes into WM989 V600E BRAF melanoma cells. Using fluorescence-activated cell sorting (FACS), we isolated 350,000 GFP-positive cells, each carrying a unique barcode. We then expanded these barcoded cells for approximately 6 doublings, yielding around 23 million cells total. To test whether resistance was heritable across these divisions, we divided the population equally across 12 treatment arms (∼1.9 million cells per arm), with two replicates of each of the six treatments (Figure 2A).

After treatment, we sequenced the barcode library from the genomic DNA of resistant cells and compared the barcode abundance between biological replicates. A high concordance between replicates would indicate that resistance is conferred by a heritable mechanism preserved across the cellular generations within a clone (Figure 2B). Consistent with this hypothesis, we found a significant odds ratio and high Jaccard similarity indices for the top 10% of clones in each treatment: dabrafenib, trametinib, CoCl_2_, acidic media, cisplatin, and doxorubicin (Figures 2C and S1B). This result demonstrates that resistance to our six different treatments is determined by heritable mechanisms that persist over approximately six doublings.

Building on previous studies showing that initial gene expression states predict clonal resistance to single treatments,^16^^,^^17^^,^^19^^,^^27^^,^^30^^,^^31^ our highly multiplexed barcoding strategy allows us to investigate whether rare gene expression states enable clones to develop resistance to multiple, or potentially all, treatments. We first identified the top 10% most abundant resistant clones per condition (∼500–1,000 clones per condition) (Figure 2D). We then asked whether resistance to treatment was related between pairs of conditions. We performed pairwise comparisons of every combination of the six treatment conditions, where we found that the top resistant clones had significant overlaps (odds ratio > 1 and *p* < 0.05) in every comparison (Figures 2E and 2F), though the magnitude varied. A sensitivity analysis across varying thresholds for clone abundance revealed that the odds ratio declines once considering more than the top 10% most abundant resistant clones per condition (Figure S1D). To provide additional global metrics of clonal concordance, we calculated Jaccard similarity indices for all pairwise treatment comparisons (Figure S1C). These analyses confirmed our odds ratio findings, with the highest similarity between dabrafenib- and trametinib-resistant clones and a distinct clustering among chemotherapeutic treatments. As expected, resistance to dabrafenib and trametinib was highly concordant, as both drugs target the MAPK signaling pathway,^48^^,^^49^^,^^50^^,^^51^ and, importantly, shows that our method can effectively identify pairs of treatments where the survival of a clone to one treatment is associated with survival to the second. Meanwhile, resistance to CoCl_2_, acid, cisplatin, and doxorubicin was highly related.

Expanding our analysis to examine overlap across all six treatments simultaneously, we found that ∼20%–40% of the top resistant clones in each condition were unique to that treatment (Figure 2G). However, the remaining top resistant clones overlapped with at least one other treatment, with 11 rare clones captured in the top 10% for all six treatments. These results reveal two distinct resistance strategies within our clonal population: “specialist” clones that develop resistance to single treatments (supporting hypothesis 1, Figure 1C) and “generalist” clones capable of resisting multiple treatments (supporting hypothesis 2, Figure 1D).

### Combining high-throughput, multi-treatment clonal tracing with scRNA-seq measures clonal gene expression before and after treatment

**Figure 3:**
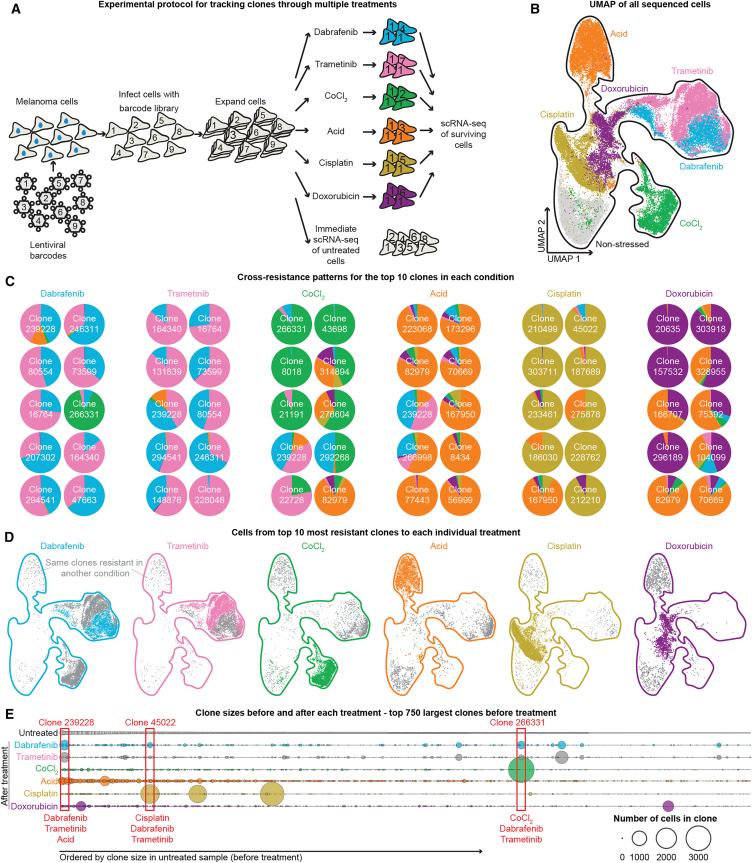
Combining multi-treatment, high-throughput clonal tracing with scRNA-seq identifies clones with multi-treatment tolerance (A) Experimental protocol for combining scRNA-seq with multi-treatment, high-throughput clonal tracing across treatment with dabrafenib, trametinib, CoCl_2_, acidic media, cisplatin, and doxorubicin. (B) UMAP of all sequenced cells from (A). Cells are colored by their treatment condition. Clustering was performed using the top 20,000 most variable genes. (C) Pie charts depicting the top 10 most resistant clones (as measured by absolute cell number in the given condition) for each individual treatment. Pie charts include both the cells from these clones in the given condition and those that were captured in another condition. Cells that were detected in other conditions were not used in determining the top 10 most resistant clones. (D) UMAP plots depicting the top 10 most resistant clones (as measured by absolute cell number in the given condition) for each individual treatment. Plots contain both the cells from these clones in the given condition and those that were captured in another condition. Cells are colored according to the specific treatment condition in which they were identified as top resistant clones, while cells from the same clones that appeared in other treatment conditions are shown in gray. Cells that were detected in other conditions were not used in determining the top 10 most resistant clones. (E) Dot plot displaying the 750 largest clones from the untreated population before treatment (gray). The size of the same clones after each treatment is displayed below. We further calculated Pearson correlations between the log_2_(number of cells + 1) for the untreated sample and each individual condition after treatment. The Pearson *r* values are as follows: 0.11 (dabrafenib), 0.07 (trametinib), CoCl_2_ (0.41), 0.71 (acid), 0.30 (cisplatin), and 0.58 (doxorubicin). See also Figures S2 and S3.

Having seen that clones can develop resistance to one or multiple treatments, we sought to measure clonal gene expression states across treatments and ultimately identify gene expression states that underlie such resistance. We designed an experiment to identify resistant clones and trace them back to their initial gene expression states (Figure 3A). By initiating all treatment arms and initial scRNA-seq measurements from the same pool of barcoded cells, we could subject all clones to each treatment while also measuring their initial gene expression state. We generated a pool of ∼200,000 uniquely barcoded cells and allowed them to grow for 20 days (∼5.5 doublings), balancing the need to maintain resistance heritability with the need to ensure adequate clone representation across treatment arms (Figure 2C). We then performed scRNA-seq on a subset of untreated cells and then split the remaining barcoded cells into six samples for different treatments. After 4 weeks, we harvested the resistant cells and performed scRNA-seq with paired barcode sequencing. From these data, we found that resistant cells from different treatments occupied distinct transcriptional states, except dabrafenib and trametinib (Figure 3B). Finally, we used barcodes to link single cells to their clonal identity, allowing us to identify cells from the same clone before and after treatment.

To ensure that the pre-treatment transcriptional states we identified were not influenced by technical artifacts, we performed batch correction on the untreated population using both Harmony and Seurat integration methods. The untreated cells were sequenced across three separate lanes, allowing us to assess batch effects. Both correction methods showed that the distinct clusters observed in the untreated population represent biological heterogeneity rather than technical variation, with key resistance markers maintaining their expression patterns after correction (Figures S1E and S1F). Applying correction across all treatment conditions resulted in overcorrection, as quantified by increased cell-type local inverse Simpson's index (LISI) and reduced inter-condition centroid distances (Figures S1G and S1H).

To investigate clonal resistance patterns to individual and multiple treatments at the single-cell level, we identified the 10 largest, most resistant clones after each treatment and visualized them in low-dimensional space (Figure 3B). We found that some cells from the 10 most resistant clones that map to a given condition had also developed resistance in other treatment arms (Figure 3C). Further analysis of individual clones in our single-cell dataset revealed that some clones predominantly developed resistance to a single treatment, while others showed multi-treatment resistance (Figure 3D).

To connect resistant clones to their pre-treatment gene expression state, we first needed to rule out the possibility that resistance arose simply from the largest clones at the initial time. We leveraged our experimental design, which included measurements of clone sizes prior to treatment, to test this hypothesis. For dabrafenib- (*r* = 0.11) and trametinib- (*r* = 0.07) treated cells, Pearson correlations of clonal size before and after treatment were very low (Figure 3E). Meanwhile, there were observable correlations of clone sizes before and after treatment for CoCl_2_ (*r* = 0.41), acid (*r* = 0.71), cisplatin (*r* = 0.30), and doxorubicin (*r* = 0.58) (Figure 3E). However, for dabrafenib-, trametinib-, CoCl_2_-, cisplatin-, and doxorubicin-treated cells, the largest resistant clones came from initial clones of variable sizes (Figure 3E). Only for acidic media treatment was the final clone size highly correlated with size before treatment and without outliers of previously small clones that became highly resistant. These data support previous findings that pre-treatment size is not the sole determinant of resistance development,^45^ allowing us to focus on identifying the gene expression states that confer future treatment resistance.

### Pre-existing cell states predict resistance to individual and multiple treatments

Before linking resistant clones to their pre-treatment gene expression states, we first verified that cells within clones maintain similar transcriptional profiles over time.^45^ We found that intra-clone gene expression similarity was significantly higher than random groupings, both before and after treatment (Figures S2A–S2G). Importantly, when we restricted our analysis to resistance-predictive genes, we observed substantially higher intra-clonal correlations compared to variable or random genes, demonstrating that clones maintain coherent expression of functionally relevant resistance mechanisms (Figures S2H–S2M). This preservation of gene expression justifies our approach of treating cells from the same clone as similar in subsequent analyses.

**Figure 4:**
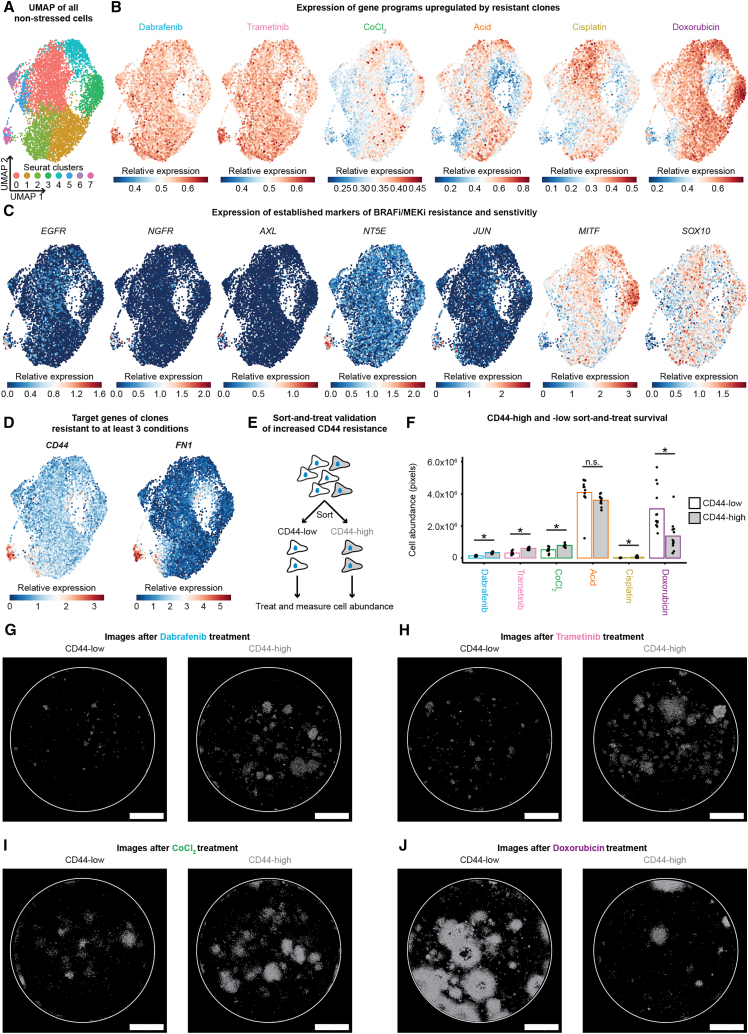
Signaling through CD44 is predictive of multi-treatment resistance (A) UMAP of all untreated cells colored by Seurat clustering using the top 20,000 most variable genes. (B) Relative expression of UCell^52^ scoring of genes upregulated in clones that will go on to develop resistance to each condition. (C) Relative expression of established markers of BRAFi/MEKi (*EGFR*, *NGFR*, *AXL*, *NT5E*, and *JUN*) and sensitivity (*MITF* and *SOX10*). (D) Relative expression of *CD44* and *FN1*, markers of resistance to at least three different treatments. (E) Experimental protocol for sort-and-treat validation of CD44 as a marker of multi-treatment analysis. (F) Results of CD44 sort-and-treat validation. Data are displayed as normalized cell abundance (in pixels). ∗*p* < 0.05 was determined by a two-sided Welch two-sample *t* test. (G–J) Representative images of binarized cell nuclei after the sort-and-treat experiment in (E) and quantification in (F). Scale bars represent 5 mm. See also Figures S3–S6 and Tables S1, S2, and S3.

We then traced the top five most resistant clones for each treatment back to their corresponding cells in the untreated population. For each founder population, we identified upregulated genes as compared to the remaining untreated cells. We classified these upregulated gene sets as being associated with developing resistance for each treatment (Table S1) and performed gene set enrichment (Figure S3A). Note that this analysis is different from a differential expression between cells before and after treatment, as our goal is to identify those genes that, before treatment, could be markers of the clones that will survive in the future. We then scored all untreated cells based on expression of these gene sets and visualized the results on the uniform manifold approximation and projection (UMAP) embedding of the untreated population only (Figures 4A and 4B).

To validate that these resistance programs predict outcomes beyond the top 5 clones, we examined the relationship between pre-treatment resistance program scores and final clone size across all resistant clones for each treatment. We found significant positive correlations for most conditions, confirming that our resistance signatures broadly predict resistance capacity (Figure S3B). Notably, when we examined the three multi-treatment-resistant clones identified in Figure 3E, each showed elevated resistance program scores specifically for the treatments to which they developed resistance, suggesting that multi-treatment resistance may arise through co-expression of multiple single-treatment resistance programs within individual clones.

Signatures predictive of dabrafenib and trametinib resistance were expressed on a spectrum across the entire untreated population, with the highest levels in cluster 7 and part of cluster 2 (Figure 4B). Conversely, cells with lower expression of these signatures showed higher expression of gene sets associated with resistance to CoCl_2_, acidic media, cisplatin, and doxorubicin, primarily in the remaining clusters. These results corroborate that resistance programs are related across treatments, but dabrafenib and trametinib are distinctly coupled separately from the other treatments (Figure 2F).

To contextualize these resistance-associated clusters, we performed pathway enrichment analysis (Tables S2 and S3). Dabrafenib and trametinib resistance-associated clusters, 2 and 7, were enriched for epithelial-mesenchymal transition (EMT) and hypoxia markers and depleted for MYC signaling. These clusters also showed higher expression of previously identified markers of resistance to BRAFi and MEKi, including *EGFR*, *NGFR*, *AXL*, *NT5E*, and *JUN*^16^^,^^19^^,^^26^^,^^45^^,^^53^^,^^54^^,^^55^^,^^56^ (Figure 4C). In contrast, the remaining clusters showed higher expression of more differentiated (melanocytic and neural crest-like) markers *MITF* and *SOX10*, often associated with susceptibility to BRAFi and MEKi.^16^^,^^20^^,^^55^^,^^57^

Notably, the dabrafenib and trametinib resistance programs were not restricted to clusters 2 and 7, which had high *EGFR*, *NGFR*, and *AXL* expression. While our previous work demonstrated that *EGFR*/*NGFR*/*AXL/NT5E*-high cells are resistant to BRAFi, we still observe some resistant colonies in *EGFR*/*NGFR*/*AXL/NT5E*-low populations, albeit at a much lower frequency.^16^^,^^19^ We thus wondered how resistance arises from these other initial state clusters. Pathway analysis on these clusters revealed enrichment in cell cycle signaling, oxidative phosphorylation, and MYC signaling. These data point to two paths of resistance to BRAFi and MEKi, including an *EGFR*/*NGFR*/*AXL/NT5E*-low population that becomes resistant and is derived from the same clusters of cells that become resistant to CoCl_2_, acidic media, cisplatin, and doxorubicin.

We next investigated expression states predictive of multi-treatment resistance. By comparing gene expression signatures associated with resistance in each condition, we identified an overlapping set of 11 genes found in at least three conditions (Figure S3C). Of particular interest, *CD44* and *FN1* were associated with resistance to dabrafenib, trametinib, and CoCl_2_ (Figure 4D). We focused on CD44 because it is a cell-surface marker described for cancer stem cells,^58^^,^^59^^,^^60^ is linked with EMT,^61^ and is associated with increased metastatic potential and decreased survival in melanoma.^62^ We also performed the same analysis tracing the top 10 most resistant clones for each treatment and found differences in the genes predictive across multiple conditions, as CD44 was not detected (Figure S4), but five genes remained in both analyses (*CTSD*, *GYPC*, *NQO1*, *SNHG29*, and *AC207130.1*).

To functionally validate CD44 as a marker in multi-treatment resistance, we used FACS to separate CD44-low and CD44-high cells and treated each population with our panel of six treatments individually for 1 month (Figure 4E). Consistent with our clonal tracing data, CD44-high cells exhibited significantly more resistance to dabrafenib, trametinib, and CoCl_2_, while CD44-low cells were more resistant to doxorubicin (Figures 4F–4J, S5A, and S5B). We also compared *CD44* to *NT5E*, which we previously found to be a marker of pre-existing resistance to BRAFi,^19^ and found that CD44-high cells were distinct from NT5E-high cells, showing a different profile of resistance (Figures S5C and S5D). To determine whether CD44 functions as a driver of resistance, we tested whether pharmacological inhibition of CD44 could sensitize cells to treatment. However, treatment with Angstrom6, a peptide inhibitor of CD44’s interaction with hyaluronic acid,^63^ did not consistently and significantly sensitize cells to dabrafenib (Figures S5E and S5F), suggesting that CD44 may function more as a marker of resistant cell states.

To identify potential molecular mechanisms underlying CD44-mediated multi-treatment resistance, we examined whether CD44-high cells exhibit classical multi-drug resistance (MDR) pathways. We performed targeted analysis of genes associated with three major MDR mechanisms: ABC transporters and efflux pumps, lysosomal sequestration and autophagy pathways, and anti-apoptotic regulators. Using odds ratio analysis, we found that CD44-high cells showed significant enrichment for several lysosomal and autophagy-related genes, with dramatically elevated odds ratios for *SRGN* (serglycin) and *VMP1* (vacuole membrane protein 1) (Figures S6A and S6B). We also observed high enrichment for *GJA1*, though this gene was expressed in very few cells overall compared to the other targets (Figures S6C–S6F).

Given this enrichment for lysosomal pathway genes, we hypothesized that CD44-high cells possess elevated lysosomal activity that contributes to their multi-treatment resistance. To test this, we co-stained cells with CD44 antibody and LysoTracker dye to simultaneously measure CD44 expression and lysosomal content.^64^^,^^65^ CD44-high cells demonstrated significantly increased lysosomal activity compared to CD44-low cells even in untreated conditions, and this difference was further enhanced upon treatment with dabrafenib, trametinib, or chloroquine across multiple time points (Figures S6G–S6I). These results suggest that pre-existing CD44-high cells have elevated lysosomal activity, which could contribute to their multi-treatment resistance, potentially through enhanced drug sequestration and degradation.

### Clonal states before treatment predict diverse resistant end states

**Figure 5:**
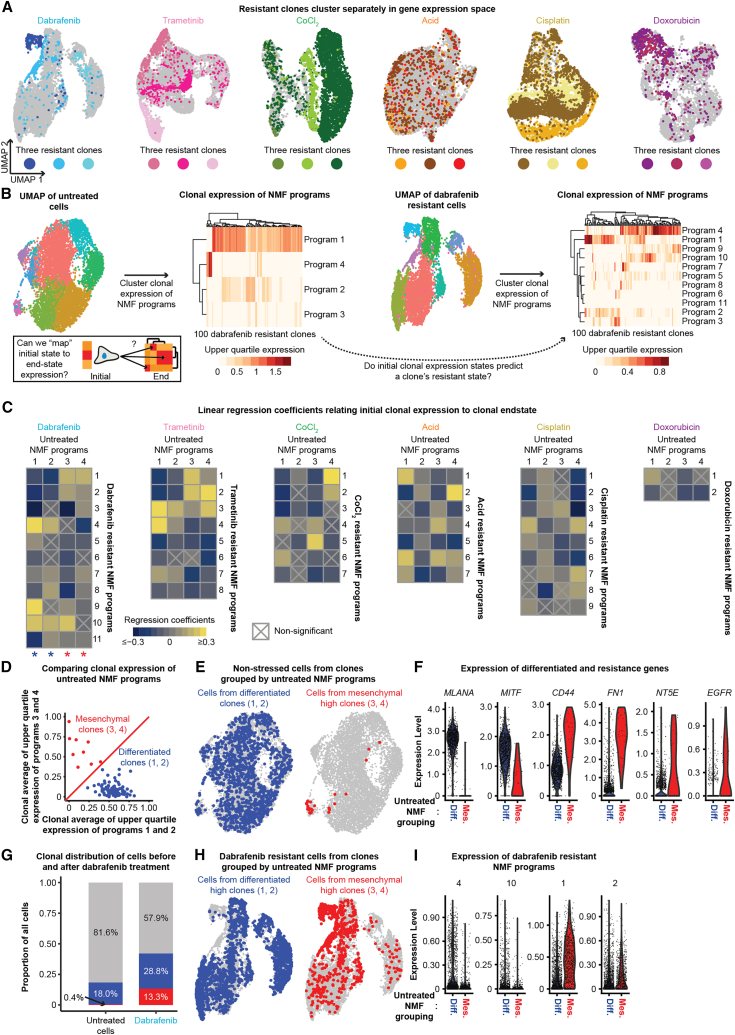
Clonal differences before treatment are predictive of resistant end states (A) Treatment-resistant UMAPs highlighting three resistant clones that separate by gene expression state. For each condition, we clustered based on the top 20,000 most variable genes. (B) Schematic for questioning whether we can map initial-state to end-state expression. For each condition individually, the top 100 most resistant clones were identified. We then performed cNMF clustering to identify gene expression signatures before and after treatment. The upper quartile of clonal expression of each NMF program was clustered. These values will be used to map between initial-state and end-state gene expression on a clone-by-clone basis. (C) Heatmaps of regression coefficients correlating untreated NMF program expression to resistant NMF programs across each condition’s top 100 most resistant clones. Gray Xs indicate that the correlation is not significant when compared to permuted correlations by a Z-test. Blue ∗ highlights the differentiated clones (untreated NMF program 1 and 2 high). Red ∗ highlights the mesenchymal clones (untreated NMF program 3 and 4 high). (D) Scatterplot of clonal average of the top quartile of untreated NMF program 1 and 2-high versus untreated NMF program 3 and 4-high expression. Red line highlights *y* = *x*. All clones below the red line are colored blue to represent differentiated clones, and all clones above the red line are colored red to represent mesenchymal clones. (E) UMAP of untreated cells highlighting cells from differentiated clones (blue, left) or mesenchymal clones (red, right). (F) Violin plots showing expression of melanocytic markers (*MLANA* and *MITF*) and markers of resistance to BRAFi (*CD44*, *FN1*, *NT5E*, and *EGFR*) across untreated cells from differentiated (Diff.) or mesenchymal (Mes.) clones. (G) Stacked colored bars represent the proportion of cells from clones that are differentiated, mesenchymal, or a part of other clones, both before and after dabrafenib treatment. The percentage of the population for each group is also displayed. (H) UMAP of dabrafenib-resistant cells, highlighting cells from differentiated clones (blue, left) or mesenchymal clones (red, right). (I) Violin plots showing expression of dabrafenib-resistant NMF programs 4, 10, 1, and 2 across dabrafenib-resistant cells from Diff. or Mes. clones. See also Figures S7–S10.

Our multi-treatment clonal tracing analysis revealed multiple clusters of untreated cells that serve as the origins of resistance to each treatment (Figure 4B). To investigate whether these distinct initial states give rise to different resistant end states, we first visualized three of the largest clones after each treatment in low-dimensional space. We observed distinct transcriptional states among the clones, particularly for dabrafenib, trametinib, CoCl2, and cisplatin, indicating that even within the same treatment arm, clones can achieve resistance through different transcriptional programs (Figure 5A). While previous work has shown diverse end states in BRAFi and MEKi,^17^^,^^45^ our approach allows us to directly investigate the relationship between specific initial cellular states and their corresponding resistant outcomes.

To formally map how initial clonal expression states relate to diverse resistant outcomes, we employed consensus non-negative matrix factorization (cNMF) clustering^66^ to identify coordinated gene programs. Importantly, because all treatment arms originated from the same pool of barcoded cells, we performed NMF clustering once on the entire untreated population, yielding four universal gene programs that could be tracked across all conditions. We then performed separate NMF clustering on each resistant population to identify treatment-specific programs (Figures 5B and S7A–S7G). We identified the NMF programs that showed variable levels across each population and calculated the upper quartile of clonal expression for these programs in the top 100 most resistant clones. We then used linear regression to compare clonal expression of the four shared untreated NMF programs with each treatment-specific resistant NMF program, assessing significance by comparing with random permutations (Figures 5C and S8). This design enables direct comparison of how the same initial transcriptional states map to different resistance outcomes across treatments.

We found significant clonal correlations between initial and resistant NMF programs for all treatments (Figure 5C). In particular, dabrafenib-resistant cells showed two distinct groupings: untreated NMF programs 1 and 2 strongly correlated with dabrafenib-resistant programs 4 and 10, while untreated NMF programs 3 and 4 correlated with dabrafenib-resistant programs 1 and 2. We classified clones as being either differentiated (from untreated NMF program 1 and 2 high, 91/100 clones) or mesenchymal (from untreated NMF program 3 and 4 high, 9/100 clones) based on their NMF program expression and location in low-dimensional space^20^ (Figures 5D and 5E).

Cells from differentiated clones expressed melanocytic markers, while mesenchymal cells expressed known markers of resistance to BRAFi, including *CD44* and *FN1* (Figures 4C, 5E, 5F, and S7A). These data suggest that both differentiated and mesenchymal cells can develop resistance to dabrafenib through distinct transcriptional mechanisms. Despite both populations developing resistance, we observed that the more mesenchymal, RTK-expressing clones have increased resistance to BRAFi, with the mesenchymal clones increasing by 33.2× (13.3/0.4 = 33.2×) during treatment, while the differentiated clones only increased by 1.6× (28.8/18.0 = 1.6×) (Figure 5G).

The predictive relationship between differentiated versus mesenchymal initial states and resistant cell fates extended across multiple treatment conditions. Analysis of trametinib and CoCl_2_ showed that differentiated and mesenchymal clones consistently occupied distinct transcriptional territories after developing resistance (Figures S9A–S9C). Notably, while dabrafenib, trametinib, and CoCl_2_ preferentially enriched mesenchymal clones, doxorubicin, acid, and cisplatin showed predominant selection of differentiated clones (Figures S9D–S9F). This result further confirms that the initial cellular differentiation state influences the specific resistance mechanisms accessible in different treatments.

To next characterize the resistant end states, we analyzed differentially expressed signaling pathways^67^^,^^68^ between the dabrafenib-resistant NMF programs 4 and 10 (originating from differentiated clones) and the dabrafenib-resistant NMF programs 1 and 2 (originating from mesenchymal clones) (Figures 5H, 5I, and S10A–S10D). Initially, mesenchymal, RTK-expressing clones exhibited higher expression of EMT and oxidative phosphorylation pathways upon developing resistance. In contrast, the more differentiated clones exhibited higher expression of KRAS-signaling-related genes after treatment, suggesting a mechanism for an alternative path to resistance.^69^ Together, these results point to a model in which resistance can emerge through divergent transcriptional pathways, each originating from a specific cellular state, rather than a universal route. More broadly, these results highlight that our framework can map these multiple paths toward resistance, which can be easily applied to other cancer types and treatments.

## Discussion

Our study addresses a critical gap in cancer resistance research by providing a framework for multi-treatment, high-throughput clonal tracing to uncover rare multi-treatment-resistant cells. While recent advances combining clonal tracing with scRNA-seq have yielded valuable insights into resistance phenotypes,^17^^,^^19^^,^^26^^,^^27^^,^^28^^,^^29^ these approaches typically focus on a single treatment. In practice, however, patients often receive multiple lines of treatment or combinations of treatment during the course of their disease.^70^^,^^71^^,^^72^^,^^73^ By simultaneously assessing cellular response across many treatments, our approach captures how tumor heterogeneity generates resistance to multiple therapeutic modalities.

The flexibility of our experimental and computational framework opens avenues for diverse cancer research applications. Future studies could extend this approach to investigate resistance to emerging immunotherapies, such as immune checkpoint inhibitors, cancer vaccines, or CAR-T therapy.^6^^,^^7^^,^^8^^,^^9^ Integration with *in vivo* models^17^^,^^29^ could elucidate how *in vitro* resistance-associated gene expression states correlate with tumor formation and treatment response in physiological contexts. Further, combining our methodology with studies of drug delivery could identify cellular states influencing the uptake of delivered targeted therapies.^74^

While our multi-treatment clonal tracing design offers improvements in multiple treatment arms, there are added challenges with the scale of these experiments. Increasing the number of treatment arms necessitates a larger initial clone population, which can limit the per-clone statistical power in the untreated population due to sequencing costs and the lack of selection at the untreated stage. As new single-cell sequencing techniques allow for higher throughput at lower cost and sequencing costs drop, these studies could be scaled to include a larger set of diverse cell types and even patient-derived models, enabling a broader and more robust exploration of the relationship between tumor heterogeneity and drug response.

Our work builds upon prior studies of multi-treatment resistance in melanoma, which have focused on specific cell-intrinsic mechanisms.^75^^,^^76^^,^^77^^,^^78^^,^^79^^,^^80^^,^^81^ By taking an unbiased view through the lens of cancer cell heterogeneity, we identified several known mechanisms of multi-treatment resistance in our system, including high levels of oxidative phosphorylation and expression of stem-like markers.^78^^,^^79^^,^^80^^,^^81^ Notably, we observed enrichment of oxidative phosphorylation^80^^,^^81^ signaling in a cluster of *MITF/SOX10*-high cells associated with resistance to CoCl_2_, acidic media, cisplatin, and doxorubicin (Figures 4B and 4C). Additionally, we identified *CD44*, an established cancer stem cell marker,^58^^,^^59^^,^^60^ as a marker of multi-treatment resistance in our study^78^^,^^79^ (Figures 4D–4J, S5A, and S5B). Our approach is distinguished from this literature by its ability to locate these programs in separate subpopulations of cancer cells within a single experiment, allowing for a direct comparison of resistance mechanisms across a selected panel of treatments (Figures 4B and 4C). Of note, we did not observe enrichment of ABC drug efflux pumps in our untreated melanoma cells, highlighting one key difference between our findings and recent literature^75^^,^^76^^,^^77^ (Table S1).

In conclusion, we demonstrate that rare clones can develop multi-treatment resistance when subjected to a panel of six treatments with varied mechanisms of action. By pairing scRNA-seq with clonal tracing, we identified gene expression states underlying multi-treatment resistance and found that clones with different initial states follow divergent paths toward resistance within a single treatment. These findings provide a framework for identifying broadly resistant clonal cancer cell states, potentially informing strategies to target and eliminate treatment resistance more comprehensively.

### Limitations of the study

Beyond the technical challenges of scale, we also acknowledge important limitations of our *in vitro* model approach. First, using an established melanoma cell line (WM989 A6-G3) may not accurately represent the full spectrum of cancer evolution, as this is just one cell line, and these cells have been selected through long-term culture conditions that could alter their resistance mechanisms compared to primary tumors. Second, our *in vitro* drug resistance models lack the complexity of the *in vivo* microenvironment, including immune cell interactions, stromal components, and metabolic gradients. The controlled culture conditions differ substantially from the heterogeneous metabolic environment of tumors, where variable oxygen levels, pH, and nutrient availability create additional selective pressures that influence drug response. While we attempted to model some of these stresses (using CoCl_2_ for hypoxia and acidic media for extracellular acidosis), these treatments only partially recapitulate the multifaceted metabolic challenges faced by tumors *in vivo*. Future studies incorporating patient-derived models and *in vivo* systems will be essential to validate these findings in more physiologically relevant contexts. Finally, while we identified CD44 as a marker of multi-treatment resistance, its predictive value will vary across cancer types and genetic backgrounds.

## Resource availability

### Lead contact

Further information and requests for resources and reagents should be directed to and will be fulfilled by the lead contact, Sydney M. Shaffer (sydshaffer@gmail.com).

### Materials availability

This study did not generate new materials.

### Data and code availability

scRNA-seq data are available at GEO: GSE279162. Code is available at Zenodo: https://doi.org/10.5281/zenodo.13935305. Raw and processed sequencing of gDNA barcodes is available at Figshare: https://doi.org/10.6084/m9.figshare.25029338 and https://doi.org/10.6084/m9.figshare.25024463. FASTQ files of clonal barcodes amplified from 10× cDNA are available at Science Data Bank: https://doi.org/10.57760/sciencedb.14654. The barcode pipeline output is available at Science Data Bank: https://doi.org/10.57760/sciencedb.14653. Time-lapse imaging of resistance formation is available at Science Data Bank: https://doi.org/10.57760/sciencedb.14740. CD44 sort and treat imaging data are available at Science Data Bank: https://doi.org/10.57760/sciencedb.14737. NT5E sort and treat imaging data are available at Science Data Bank: https://doi.org/10.57760/sciencedb.14724. Any additional information required to reanalyze the data reported in this paper is available from the lead contact upon request.

## Acknowledgments

We thank the members of the Shaffer Lab for input on the experiments and figures of the manuscript. We thank the Arjun Raj Lab at the University of Pennsylvania for the barcoding plasmid library. A.J.F., P.E.W., and S.M.S. recognize support from Grants for Faculty Mentoring Undergraduate Research (A.J.F. and S.M.S., 2021, and P.E.W. and S.M.S., 2022). S.M.S. recognizes support from the Wistar/10.13039/100006920Penn SPORE (P50 CA261608) and 10.13039/100000002NIH Director’s Early Independence Award (DP5OD028144). S.M.S. and N.R.Z. recognize support from the NIH (R01GM149671).

## Author contributions

Conceptualization, D.L.S. and S.M.S.; methodology, D.L.S., P.E.W., G.E.W., C.J.C., and S.M.S.; software, D.L.S., P.E.W., and K.Z.L.; formal analysis, D.L.S., P.E.W., C.J.C., and G.E.W.; investigation, D.L.S., P.E.W., C.J.C., and G.E.W.; data curation, D.L.S., P.E.W., C.J.C., and A.J.F.; writing – original draft, D.L.S., P.E.W., and S.M.S.; writing – review & editing, D.L.S., P.E.W., K.Z.L., A.J.F., N.R.Z., C.J.C., and S.M.S.; visualization, D.L.S., P.E.W., and S.M.S.; funding acquisition, S.M.S. and P.E.W.; supervision, S.M.S.

## Declaration of interests

The authors declare no competing interests.

## Declaration of generative AI and AI-assisted technologies in the writing process

During the preparation of this work, the authors used Claude to check the writing for proper grammar and spelling and to perform some editing of the writing. After using the tool, the authors reviewed and edited the content as needed and take full responsibility for the content of the publication.

## STAR★Methods

### Key resources table

**Table:** 

REAGENT or RESOURCE	SOURCE	IDENTIFIER
**Antibodies**		

PE anti-mouse/human CD44 (Clone IM7)	BioLegend	Cat# 103007-BL; RRID: AB_312958
APC anti-human CD73 (NT5E) (Clone AD2)	BioLegend	Cat# 344005; RRID AB_1877158
APC anti-mouse/human CD44 (Clone IM7)	BioLegend	Cat# 103011; RRID: AB_312962

**Chemicals, peptides, and recombinant proteins**		

Dabrafenib	Cayman Chemical	Cat# 16989-10
Trametinib	Cayman Chemical; SelleckChem	Cat# 16292-50; Cat# 871700-17-3
Cobalt(II) chloride (CoCl_2_)	Spectrum Chemical	Cat# 18609836
Cisplatin	Cayman Chemical	Cat# 13119-100
Doxorubicin	Tocris Bioscience	Cat# 25316-40-9
Angstrom6 (A6 peptide)	MedChem Express	Cat# HY-P2230
Chloroquine diphosphate	TCI America	Cat# C2301100G
LysoTracker Green DND-26	Invitrogen	Cat# L7526
Polybrene	MilliporeSigma	Cat# TR1003G
MES buffer (1 M, pH 5.0)	Thermo Scientific	Cat# AAJ61960AK
Formaldehyde (4%)	Fisher Scientific	Cat# BPBP531500
OPTI-MEM	Gibco	Cat# 31985062
PEI (Polyethylenimine)	Polysciences	Cat# 23966-1
AMPure XP magnetic beads	Beckman Coulter	Cat# A63881
Propidium iodide	Tocris Bioscience	Cat# 51-351-0

**Critical commercial assays**		

Chromium Next GEM Single Cell 3′ Kit v3.1 Dual Index	10× Genomics	Cat# PN-1000269
QIAamp DNA Mini Kit	Qiagen	Cat# 51304
Agilent Bioanalyzer High Sensitivity DNA Kit	Agilent	Cat# 5067-4626
Qubit dsDNA High Sensitivity Assay	Invitrogen	Cat# Q32854
NEBNext Q5 HotStart HiFi PCR Master Mix	New England Biolabs	Cat# M0543S
NextSeq 500/550 Mid Output Kit v2.5 (150 cycles)	Illumina	Cat# 20024904
NextSeq 500/550 High Output Kit v2.5 (75 cycles)	Illumina	Cat# 20024906

**Deposited data**		

scRNA-seq data	This paper	GEO: GSE279162
Analysis code	This paper	Zenodo: https://doi.org/10.5281/zenodo.13935305
Raw gDNA barcode sequencing data	This paper	Figshare: https://doi.org/10.6084/m9.figshare.25029338
Processed gDNA barcode data	This paper	Figshare: https://doi.org/10.6084/m9.figshare.25024463
Clonal barcoding FASTQ files	This paper	Science DataBank: https://doi.org/10.57760/sciencedb.14654
Barcode pipeline output	This paper	Science DataBank: https://doi.org/10.57760/sciencedb.14653
Time-lapse imaging of resistance formation	This paper	Science DataBank: https://doi.org/10.57760/sciencedb.14740
CD44 sort and treat imaging data	This paper	Science DataBank: https://doi.org/10.57760/sciencedb.14737
NT5E sort and treat imaging data	This paper	Science DataBank: https://doi.org/10.57760/sciencedb.14724

**Experimental models: Cell lines**		

WM989 A6-G3 melanoma cells	Shaffer et al.^16^; Schaff et al.^45^	N/A
WM989 A6-G3 EGFP-6xMYCNLS melanoma cells	This paper	N/A
HEK293T	ATCC	CRL-3216

**Oligonucleotides**		

Primers for barcode amplification from gDNA and 10× cDNA	This paper; Harmange et al.^19^; Goyal et al.^17^	See Table S4

**Recombinant DNA**		

Rewind barcode plasmid library	Emert et al., 2021	N/A
VB211026-1265ped (EFS-EGFP-6xMYCNLS)	VectorBuilder	https://en.vectorbuilder.com/vector/VB211026-1265ped.html
pMD2.G (VSVG)	Addgene	Addgene# 12259
psPAX2	Addgene	Addgene# 12260

**Software and algorithms**		

Cell Ranger v5.0.1	10× Genomics	https://support.10xgenomics.com/single-cell-gene-expression/software/pipelines/latest/what-is-cell-ranger
Seurat v4	Hao et al.^82^	https://satijalab.org/seurat/
Scanpy	Wolf et al.^83^	https://scanpy.readthedocs.io/
UCell v2.0.1	Andreatta and Carmona^52^	https://github.com/carmonalab/UCell
cNMF	Kotliar et al.^66^	https://github.com/dylkot/cNMF
fgsea v1.22.0	Korotkevich et al.^67^	https://github.com/ctlab/fgsea
Harmony	Korsunsky et al.^84^	https://github.com/immunogenomics/harmony
scikit-image v0.21.0	van der Walt et al.^85^	https://scikit-image.org/
R	R Core Team	https://www.r-project.org/
Python	Python Software Foundation	https://www.python.org/
FlowJo	BD Biosciences	https://www.flowjo.com/
BarcodeAnalysis pipeline	Harmange et al.^19^	https://github.com/SydShafferLab/BarcodeAnalysis
venn (R package)	Dusa^86^	https://github.com/dusadrian/venn

**Other**		

Incucyte S3 live-cell imaging system	Sartorius	N/A
MoFlo Astrios cell sorter	Beckman Coulter	N/A
NextSeq 500 sequencer	Illumina	N/A
Chromium Controller	10× Genomics	N/A
Nikon Eclipse Ti2 microscope	Nikon	N/A
BD Accuri C6 flow cytometer	BD Biosciences	N/A
FACS cell strainer	Falcon	Cat# 352235

### Experimental model and study participant details

#### Cell lines and tissue culture

All experiments in this manuscript used WM989 A6-G3 melanoma cell lines. WM989 A6-G3 EGFP-6xMYCNLS cells used in time-lapse microscopy included a nuclear-localized EGFR from vector VB211026-1265ped that was constructed and packaged by VectorBuilder. This vector utilizes an EFS promoter and six repeats of MYC’s NLS to express and localize EGFP to the nucleus. Additional information can be found at https://en.vectorbuilder.com/vector/VB211026-1265ped.html. WM989 A6-G3 cells were bottlenecked as single cells twice from WM989s cells. We authenticated and validated WM989 A6-G3 cells as mycoplasma negative.^19^ We used TU2% media with 78% MCDB 153, 20% Leibovitz L-15, 2% FBS, 1.68 mM CaCl_2_, 50 U/mL penicillin, and 50 μg/mL streptomycin to culture all WM989 A6-G3 cells. Untreated cells were passaged using 0.05% trypsin-EDTA, while resistant cells were passaged using 0.25% trypsin-EDTA. HEK293T cells were used for lentivirus packaging and cultured in DMEM with 10% FBS, 50 U/mL penicillin, and 50 μg/mL streptomycin and passaged with 0.05% trypsin-EDTA. All cells were incubated at 37°C and 5% CO_2_.

For imaging, cells were fixed by washing once with 1× PBS (Invitrogen, AM9625), fixed with 4% formaldehyde in 1× PBS (Fisher, BPBP531500) for 10 min, washed twice more with 1× PBS, and kept in 70% ethanol at 4°C until needed.

### Method details

#### Treatments

We made stocks of dabrafenib at 500 μM (Cayman, 16989-10), trametinib at 5 μM (Cayman, 16292-50 and SelleckChem, 871700-17-3), CoCl_2_ at 25 mM (Spectrum Chemical Manufacturing Corporation, 18609836), cisplatin at 2 mM (Cayman,13119-100), and doxorubicin at 50 μM (Tocris Bioscience, 25316-40-9). Dabrafenib, trametinib, and doxorubicin were reconstituted in DMSO. CoCl_2_ was reconstituted in nuclease-free water. Cisplatin was reconstituted in 145 mM NaCl. For experiments, we diluted all agents in TU2% to a concentration of 1 μM dabrafenib, 10 nM trametinib, 200 μM CoCl_2_, either 5 or 15 μM cisplatin, and 50 nM doxorubicin unless described otherwise. Acidic media was made by bringing TU2% to 6.25 pH using MES buffer at 1 M, pH 5.0 (Thermo Scientific, AAJ61960AK).

#### Time-lapse imaging of colony formation

We plated 25,000 WM989 A6-G3 EGFP-6xMYCNLS per well of two six-well plates (Figure 1 A-B; Figure S1). We treated two wells per condition with 1 μM dabrafenib, 10 nM trametinib, 200 μM CoCl_2_, 6.25 pH acidic media, 15 μM cisplatin, and 50 nM doxorubicin. We replaced media with treated media every three to four days for dabrafenib, trametinib, CoCl_2_, and acidic media treated cells. Cisplatin and doxorubicin treated cells received three days of treatment followed by media changes with non-treated media. Cells were imaged once daily for 30 days on an Incucyte S3 (Sartorius) in brightfield and GFP with 300 msec of exposure time using a 4× objective.

#### Lentiviral packaging

Barcodes from the Rewind library were packaged into lentivirus as described previously.^26^^,^^45^ Briefly, HEK293T cells were cultured in 10 cm plates until being close to confluent. Per plate, we combined 500 μL of OPTI-MEM (Gibco, 31985062) with 80 μL of PEI (Polysciences, 23966-1) in a tube while combining 500 μL OPTI-MEM with 10 μg barcode plasmid, 5 μg VSVG (pMD2.G, Addgene #12259), and 7.5 μg psPAX2 (Addgene #12260) in a separate tube. We incubated the two tubes for five minutes at room temperature and combined them. We vortexed the combined solution and incubated for an additional 15 min at room temperature. We then added the mixed solution dropwise to the 10 cm plates. We incubated the plates at 37°C for 6 h before replacing the media with seven mL of fresh DMEM. We waited 24 h and verified that the cells had begun expressing GFP before starting to collect virus-containing media. We collected and replaced media every 24 h for three days, storing the virus-containing media at 4°C. We centrifuged the collected media at 3000 RPM and collected the supernatant. We filtered the virus-containing media through a 0.45 μm filter (Millipore Sigma, SE1M003M00). The filtered media was divided into 1 mL aliquots and stored at −80°C.

#### Lentiviral transduction

We transduced WM989 A6-G3 cells with barcode virus as previously described.^45^ In brief, we seeded 300,000 cells per well of six-well plates. We added 32 μL of freshly thawed virus per well with a goal of 20–25% infection efficiency in addition to 8 μg polybrene (MilliporeSigma, TR1003G). We centrifuged the plates for 25 min at 1750 RPM and incubated at 37°C overnight. In the morning, we washed the cells with DPBS and replaced the media with TU2% media.

#### Fluorescence-activated cell sorting (FACS) of barcoded cells

We isolated cells that had received a barcode during transduction as previously described.^45^ We waited three days after transducing cells with GFP-barcode virus before trypsinizing (0.05% trypsin-EDTA) cells into a single-cell suspension. We neutralized the trypsin with TU2% and washed the pellet with 1% BSA-DPBS. We resuspended the pellet with 1% BSA-DPBS containing DAPI. We filtered the cell suspension by passing it through a FACS cell strainer (Falcon, 352235). Using a Beckman Coulter MoFlo Astrios sorter with a 100 μm nozzle, we isolated barcode-expressing cells by gating for droplets that contained live singlets that were GFP-positive.

#### High-throughput, multi-clonal treatment to answer whether clonal resistance to treatment is deterministic

We used data from an experiment previously published by our group.^45^ In brief, we introduced our barcode library into the WM989 A6-G3 cell line as described previously (see “lentiviral transduction”). We isolated 350,000 uniquely barcoded cells using FACS. We then allowed the barcoded cells to proliferate for roughly 6.5 doublings. We then divided the population across 12 treatment arms, two per treatment condition. We then treated the populations with either: dabrafenib (1 μM for four weeks), trametinib (10 nM for four weeks), CoCl_2_ (200 μM for four weeks), acidic media (pH 6.25 for four weeks), cisplatin (5 μM for two weeks followed by two weeks of untreated TU2%), and doxorubicin (50 nM for 2.5 weeks followed by 1.5 weeks of untreated TU2%). Media changes were performed every three to four days. At the end of the four week treatment period, the surviving, resistant cells were trypsinized and stored at −80°C. We then isolated the barcodes from the genomic DNA (gDNA) (see “DNA barcode recovery from genomic DNA”). For each of the 12 samples, we input two replicates of 250 ng of gDNA into a PCR reaction with 25 cycles of amplification. We cleaned these samples with a double-sided bead cleanup and made an equimolar pool of all 12 samples before sequencing (see “DNA barcode recovery from genomic DNA”).

#### DNA barcode recovery from genomic DNA

We recovered barcodes from gDNA and made sequencing libraries as previously described.^26^^,^^45^ Briefly, we removed cell pellets that were stored at −80°C. If being used, we then added a “ladder” of cells with a known number and sequence (two sets of barcodes at 50, 500, and 1000 cells per barcode).^45^ We then used the QIAmp DNA Mini Kit (Qiagen, 51304) spin protocol for DNA purification from blood or bodily fluids to isolate gDNA from cells. We performed a PCR reaction using primers from Harmange et al.^19^ that contained Illumina adapter sequences, sample indices, and variable length base pair staggers to amplify the DNA barcodes from the gDNA. We used 250 ng of input gDNA (described per condition), 500 nM primers, 25 μL of NEBNext Q5 HotStart HiFi PCR MAster Mix (NEB, M0543S), and nuclease-free water to bring to a final reaction volume of 50 μL. We performed PCR reactions using a thermal cycler with settings as follows: 30 s at 98°C, 25 cycles of 10 s at 98°C and 40 s at 65°C, and five minutes at 65°C. We cleaned the amplified barcodes using a double-sided bead cleanup with AMPure XP magnetic beads (Beackman Coulter, A63881). We added 0.6× (20 μL) of beads, carried over the supernatant, added 1.2× (30 μL) of beads, washed with 80% ethanol twice, and eluted in 20 μL nuclease-free water. We measured the size of the libraries using the Agilent Bioanalyzer High Sensitivity DNA Kit (Agilent, 5067-4626) and the concentration using the Qubit dsDNA High Sensitivity Assay (Invitrogen, Q32854). We diluted and pooled libraries before sequencing using a 150 cycle mid output kit (Illumina, 20024904) on a NextSeq 500 with eight cycles for each index and 151 cycles for read one to measure the clonal DNA barcodes.

#### Multi-treatment, high-throughput clonal tracing paired with scRNA-seq

We barcoded WM989 A6-G3 melanoma cells (see “lentiviral transduction”) and sorted 200,000 uniquely barcoded cells into one well of a six-well plate (Figure 3A). We allowed cells to grow for 20 days, which translated to ∼4.88 doublings (∼5,900,000 cells as counted by a hemocytometer). For each treatment arm, we plated 737,500 cells evenly across three 10 cm plates (∼245,000 cells per plate). The remaining cells were immediately used for scRNA-seq library preparations using the 10× Genomics 3′ v3.1 Dual index Single-Cell gene expression kit (10× Genomics, PN-1000269) with a 10× Genomics Chromium Controller. Libraries were quantified using an Agilent Bioanalyzer high Sensitivity DNA Kit (Agilent, 5067-4626) and a Qubit dsDNA High Sensitivity Assay (Invitrogen, Q32854). We sequenced the samples as a 4 nM pool using a 75 cycle high output kit (Illumina, 200249606) on a NextSeq 500 with 28 cycles allocated to read one, 43 cycles allocated to read two, and 10 cycles for each index.

The day following seeding, we began treatment on each treatment arm as follows: dabrafenib at 1 μM for four weeks, trametinib at 10 nM for four weeks, CoCl_2_ at 200 μM for four weeks, acidic media at 6.25 pH for four weeks, cisplatin at 5 μM for two weeks followed by untreated TU2% for three weeks, and doxorubicin at 50 nM for two weeks followed by untreated TU2% for three weeks. After treatment was finished in each treatment arm, we performed scRNA-seq as described above.

#### DNA barcode recovery from scRNA-seq

We recovered the DNA barcodes from the full-length cDNA that was leftover from the 10× Genomics workflow as previously described.^19^^,^^45^ Briefly, we amplified the barcodes from the leftover full-length cDNA using custom primers from Goyal et al.^17^ and Harmange et al.^19^ that include illumina adapter sequences, sample indices, and variable-length base pair staggers (Table S4). We performed PCR reactions using 5 μL full-length cDNA, forward and reverse primers at 500 nM, 25 μL of NEBNext Q5 HotStart HiFi PCR Master Mix (NEB, M0543S), and nuclease-free water to bring the final reaction volume to 50 μL. We performed PCR on a thermal cycler with the following settings: 30 s at 98°C, 14 cycles of 10 s at 98°C and two minutes at 65°C, and five minutes at 65°C. We cleaned the amplified material by adding AMPure XP magnetic beads (Beckman Coulter, A63881) at 0.7× (35 μL). We washed then removed the supernatant and washed the beads twice with 80% ethanol. We then eluted the final libraries in 20 μL of nuclease-free water. We quantified the size of the libraries were using an Agilent Bioanalyzer High Sensitivity DNA Kit (Agilent, 5067-4626) and the concentration of the libraries using a Qubit dsDNA High Sensitivity Assay (Invitrogen, Q32854). We sequenced an equimolar pool of all samples with a 150 cycle mid output kit (Illumina, 20024904) on a NextSeq 500 with 28 cycles allocated to read one to capture the 10× cell barcode and UMI, 123 cycles allocated to read two to capture the clonal DNA barcodes, and eight cycles allocated to each index.

#### Sort-and-treat validation of markers of multi-treatment resistance

We collected melanoma cells in a single-cell suspension with 0.05% trypsin-EDTA. We then washed the cells once with 0.1% BSA-DPBS. We stained the cells with the CD44 antibody (1:200 dilution, PE IM7 clone, Biolegend, 103007-BL) or NT5E antibody (1:200 dilution, APC AD2 clone, Biolegend, 344005) in the dark for one hour. Then, we washed the cells twice with 0.1% BSA-DPBS before passing the cells through a cell strainer (Falcon, 352235) and added DAPI. We then sorted out the cells with the top ∼2% and bottom 5% expression of CD44 or NT5E. in 12-well plates, we then plated wells of CD44/NT5E-high and CD44/NT5E-low cells at 7000 cells/well to be fixed the day following seeding to serve as controls for the number of cells plated per group. We then took the remaining cells and plated as many wells as possible at 7000 cells/well in 12-well plates for treatment with our six different treatments. We then treated the CD44/NT5E-high and CD44/NT5E-low cells as follows: dabrafenib at 1 μM for four weeks, trametinib at 10 nM for four weeks, CoCl_2_ at 200 μM for four weeks, acidic media at 6.25 pH for four weeks, cisplatin at 15 μM for three days followed by untreated TU2% for the remainder of the four weeks, and doxorubicin at 50 nM for three days followed by untreated TU2% for the remainder of the four weeks. We then formaldehyde fixed cells and stained them with DAPI. We took 8 × 8 scans at 4× magnification with 1 s exposure using a filter set for DAPI on a Nikon Eclipse Ti2 microscope.

#### Lysotracker experiments

We performed this experiment using WM989 A6-G3 melanoma cells. We made stock solutions of dabrafenib at 500 μM (Cayman, 16989-10), trametinib at 5 μM (Cayman, 16292-50), chloroquine diphosphate at 5 mM (TCI America, C2301100G), and LysoTracker Green at 1 mM (Invitrogen, L7526). We reconstituted dabrafenib and trametinib in DMSO and chloroquine diphosphate in water. For experiments, we diluted all agents in TU2% media to final concentrations of 1 μM dabrafenib, 10 nM trametinib, 50 μM chloroquine diphosphate, and 200 nM LysoTracker.

Cells were plated at 50,000 per well in 24-well plates with three replicates per condition. Time points were collected at 24, 48, and 72 h post-treatment, and one plate served as single-stain controls for unstained, propidium iodide, CD44 antibody, and LysoTracker conditions. After adding LysoTracker in warmed media for 1-h incubation, we collected and washed cells with 0.1% BSA in PBS. Staining with APC anti-CD44 antibody [Clone IM7] (Biolegend, 103011) proceeded for 45 min on ice in darkness. Following another wash step, we analyzed cells on a BD Accuri C6 flow cytometer, gating live, single cells by size and using propidium iodide (Tocris Bioscience, 51-351-0) to exclude dead cells. We calculated geometric mean FITC measurements (LysoTracker) for cells in the top 2% and bottom 5% of CD44 expression using FlowJo.io software and assessed statistical significance using paired two-tailed t-tests with *p* < 0.05.

#### A6 inhibitor experiments

We made stocks Angstrom6 at 10 μM (MedChem Express, HY-P2230). For the experiments, we diluted all agents in TU2% to a concentration of 1 μM dabrafenib alone, or 150 nm dabrafenib and 5 nm trametinib together, and 50 nm Angstrom6. The Angstrom6 toxicity control experiment used Angstrom6 diluted to 10 nM, 25 nM, 50 nM, 75 nM, and 100 nM. Cells were plated at 20,000 cells per well into 12-well and 6-well Corning Falcon tissue culture plates. Experiments measuring confluence had measurements taken on a Sartorius Incucyte S3 in brightfield using a 4× objective. Experiments measuring nuclear area had measurements taken of formaldehyde fixed cells stained with DAPI by a Nikon Eclipse Ti2 microscope using a 4× objective. We contrasted and binarized images taken on the microscope using custom python scripts as described previously.^45^ Control plates were imaged after 4 days, and experimental conditions were imaged at the end of 35 days. Differences in cell growth were tested for significance using a paired two-tail *t* test with *p* < 0.5.

### Quantification and statistical analysis

#### Quantification of cell abundance from imaging

We contrasted and binarized images taken on the microscope and incucyte using custom python scripts as described previously.^45^ The following processing was performed using GFP scans for assessing growth during treatment (Figures 1A and 1B; Figure S1) and using DAPI scans for assessing resistance after sorting and treating (Figures 3E–3J; Figures S5A–S5D) In brief, we cropped the images to include only the inside of the well in each square image. Then, we used the scikit-image (v0.21.0)^85^ package to perform Gaussian and median filtering which smoothed the local brightness within the images and then Niblack thresholding to establish local thresholds for areas covered by cells. We binarized the filtered images by assigning any pixel where the fluorescence was greater than the local threshold ∗ (1.01–1.02) to be part of a cell (value of 1) and other pixels to background (value of 0). To remove small aberrant patches which passed filtering, we removed objects smaller than 64 pixels. Finally, we removed the outer ring of pixels which had passed thresholding but were representative of edge effects. We visually inspected the resulting images to confirm the pixels above threshold were accurately representing areas of the dish covered by cells and thus, estimating cell number. With the correctly binarized images, we quantified the number of pixels above threshold for further processing. This workflow made it possible to accurately quantify cell growth in highly confluent resistant clones that have lost contact inhibition.

For the time-lapse microscopy (Figures 1B; S1A), we then used custom R scripts to plot cell abundance over time. For imaging of sorted cells after treatment (Figures 3E–3J; Figures S5A–S5D), we normalized growth of CD44/NT5E-high and CD44/NT5E-low cells based on the abundance of plating controls that were fixed the day after treatment, as described previously.^45^ We then plotted the normalized growth after treatment between CD44/NT5E-high and CD44/NT5E-low cells and assessed whether differences were significant by a two-sided Welch two sample *t* test with *p* < 0.05.

#### Computational analysis of high-throughput, multi-treatment clonal tracing to answer whether clonal resistance to treatment is deterministic and whether clones develop resistance to multiple treatments

We performed the following analyses using custom R scripts. First, we combined the two sequencing replicates for every sample. We then performed reads per million (RPM) normalization to account for sequencing depth on all clones that had at least one read between the two biological replicates. We then plotted scatterplots and calculated the Pearson correlation of the log_2_(RPM+1) between the two biological replicates for each treatment condition (Figure 2C).

Knowing that resistance was correlated between the two biological replicates per treatment condition, we then combined the biological replicates by taking the average RPM between the samples. We then plotted rank-ordered bar plots of the average barcode abundance of all clones with RPM >1 in that condition, coloring the top 10% most abundant in each condition (Figure 2D). We then built contingency tables for every pairwise combination of treatments where the “universe” was all clones that were RPM >1 in at least one condition. Of those 17593 clones that were detected in at least one condition, we then looked at overlap between the top 10% most resistant clones per condition to build the rest of the contingency table (Figure 2E). We then calculated odds ratios and *p* values using fisher.test (Figure 2F). We then plotted the log_2_(odds ratios) in a heatmap.

Finally, we used the venn package in R (https://github.com/dusadrian/venn) to create a venn diagram of the top 10% most resistant clones for every treatment (Figure 2G).

#### scRNA-seq analysis

We used the CellRanger software version 5.0.1 (10× genomics) to generate count matrices of gene expression using the 2020-A hg38 reference genome, as previously described.^45^ We then used the Seurat v4 package^82^ to perform downstream analyses. When generating count matrices, we merged the sequencing of clonal barcodes with scRNA-seq data on a cell-by-cell basis using the CellRanger Feature Barcode technology. On a sample-by-sample basis, we removed low-quality cells that had high RNA count (likely doublets), few detected genes (likely dying cells or ambient RNA), and high proportions of mitochondrial reads (likely dying cells). We then used the following functions in Seurat to process our data: NormalizeData, FindVariableFeatures, ScaleData, RunPCA, FindNeighbors, FindClusters, and RunUMAP. We then performed additional analyses as described in their respective STAR Methods sections.

We identified differentially expressed gene sets in each cluster of the untreated population by identifying up and downregulated genes using the FindMarkers function in Seurat.^82^ We then performed GSEA^87^ using the fgsea^67^ (1.22.0) package with the Molecular Signatures Database hallmark gene sets.^68^

#### Batch correction assessment

To assess the impact of batch effects and batch correction on our dataset, we performed integration using both Harmony^84^ (RunHarmony, group.by.vars = seq_rep) and Seurat v4^82^ CCA-based integration (FindIntegrationAnchors, IntegrateData) on the untreated cells (naive lanes 1–3) and on the full dataset (all treatment conditions). We evaluated batch mixing and biological conservation using the Local Inverse Simpson’s Index (LISI),^84^ computed on the first 20 principal components of each embedding with a perplexity of 30. Integration LISI (iLISI), computed over sequencing lane labels, measures how well batches are mixed, with higher values indicating better mixing. Cell-type LISI (cLISI), computed over biological condition labels, measures conservation of biological structure, with values near 1 indicating distinct conditions are preserved. To quantify changes in inter-condition separation, we computed condition centroids in each embedding space and calculated pairwise Euclidean distances between centroids.

#### Combining scRNA-seq and barcode data

We used a pipeline published by Harmange et al.^19^ to identify clonal barcodes present in our sequencing data (https://github.com/SydShafferLab/BarcodeAnalysis). In brief, this pipeline uses FASTQ files with barcode sequencing data from 10× cDNA and/or gDNA as input material. The pipeline identifies sequences that belong to DNA barcodes and combines barcode sequences that have a Levenshtein distance of eight or less. These highly similar barcodes likely originated from the same lentiviral integration, but received alterations such as point mutations over the course of treatment or result from errors in PCR or sequencing. The pipeline then provides FASTQs that are altered to contain the corrected barcodes. The output FASTQ files for barcodes amplified from 10× cDNA are linked to the 10× Genomics cellular barcode as described in “scRNA-seq analysis”. Finally, the pipeline yields the total number of sequencing reads per barcode from all samples input into the pipeline, including those from 10× cDNA or gDNA.

We often found that individual cells had sequencing reads from more than one barcode. This could occur from a cell receiving multiple different barcodes at the time of transduction, or the capture of ambient RNA molecules during droplet generation. We used code written by Dr. Kevin Lin and Dr. Nancy Zhang that is a part of a manuscript in preparation to computationally approximate whether a cell had truly received the multiple different barcodes and combine these barcodes or to filter out ambient RNA signals. This code will be made available and this study will be cited at the time of publication.

#### Clone similarity

We assessed the transcriptional similarity of clones similar to previously described.^45^ Briefly, we analyzed clones with at least 15 cells either before or after any of the six treatments. In the clones for each condition, we found the pairwise Pearson correlation of the scale.data^82^ across the 2000 most variably expressed genes in that condition. We then compared the clonal averages of the pairwise Pearson correlations to those of randomly simulated, size-matched clones from the same condition over 100 simulations. We used a one-sided Wilcoxon Rank-Sum Test to answer whether the real clones were more similar than the simulated clones. In Figure S2, we plotted the first simulation next to the real data.

#### Identifying clonal gene expression states that are associated with resistance to one or multiple treatments

To identify clonal gene expression states that are associated with resistance to each treatment individually, we began by analyzing each treated population. In each treated population, we found the top five largest clones. We then took these five clones and identified any cells represented in the untreated population. We then used the Seurat^82^ FindMarkers function to identify genes upregulated in these cells with an avg_log2FC ≥ 0.25 and p_val ≤0.05. These upregulated genes constituted the gene sets that are associated with resistance to each treatment. We then used the UCell package (v2.0.1)^52^^,^^82^ to calculate scores of aggregated expression of each gene set for all single cells in the untreated population.

To identify genes that are associated with resistance to multiple treatments, we cross-referenced the gene sets that were associated with each treatment individually. We identified 11 genes that were associated with resistance to at least three different treatments.

#### Consensus NMF clustering and mapping of initial gene expression state to end state and stratifying different paths toward resistance to dabrafenib resistance

We converted our Seurat^82^ objects into Scanpy^83^ objects to perform consensus non-negative matrix factorization (cNMF) clustering in python. We used the cNMF package to find NMF programs across 200 iterations using the 5000 most over-dispersed genes.^66^ We performed this analysis separately for the unstressed population and each of the six resistant populations. For each population, we chose a number of components (K) that resulted in a high stability and low error. We then filtered the data to remove signal with high mean distance to k nearest neighbors. We then output the single-cell program usage scores, z-scored gene spectra scores per gene expression program, and the lists of the top 100 most important genes per program. Finally, we identified the NMF programs that had variable expression across the population for further analyses.

For the NMF mapping in Figure 5C, we loaded the single cell gene expression program usages for each individual treatment condition and the unstressed cells in R. For each individual treatment condition, we identified the 100 largest, most resistant clones for the condition that were also detected with at least two cells in the unstressed population. For each clone, we calculated the upper-quartile expression of each gene expression program in both the given treatment resistant dataset and the unstressed population. We then z scored these values and began performing linear regressions using the *lm* function. For each treatment, we performed a linear regression across all four of the clonal unstressed gene expression programs for each resistant program individually (schematized in Figure S8). We measured the regression coefficients of each unstressed program for the resistant program to understand which programs were the most predictive of the end-state program across the top 100 resistant clones. We assessed the significance of these regression coefficients by comparing them to the distribution of regression coefficients calculated by randomizing the identity of the unstressed clonal populations. We used a Z test with *p* < 0.05 to show which associations were greater than randomly simulated clones.

Finally, we loaded the z-scored gene spectra scores per gene expression program and used these values to perform GSEA^87^ on hallmark gene sets^68^ using fgsea^67^ (1.22.0) to identify enriched and depleted gene sets in each gene expression program (Figure S10).

## Supplemental information

Document S1. Figures S1–S10

Table S1. Upregulated genes predictive of survival to each individual treatment, related to Figure 4

Table S2. FindMarkers results for each untreated cluster, related to Figure 4

Table S3. GSEA on markers of untreated clusters, related to Figure 4

Table S4. Primers used for amplifying barcodes out of gDNA and 10× cDNA, related to STAR Methods

Document S2. Article plus supplemental information
