# Supplementary material for: Pre-existing cell states predict resistance to multiple treatments
Source: Cell Genom. 2026 Mar 30;6(6):101191. doi: 10.1016/j.xgen.2026.101191 (PMC13261651; doi:10.1016/j.xgen.2026.101191)
Supplement: Document S1. Figures S1–S10 [file mmc1.pdf]

Cell Genomics, Volume *6*

Supplemental information

Pre-existing cell states predict resistance to multiple treatments

Dylan L. Schaff, Phoebe E. White, Christopher J. Cote, Grace E. Watterson, Kevin Z. Lin, Aria J. Fasse, Nancy R. Zhang, and Sydney M. Shaffer

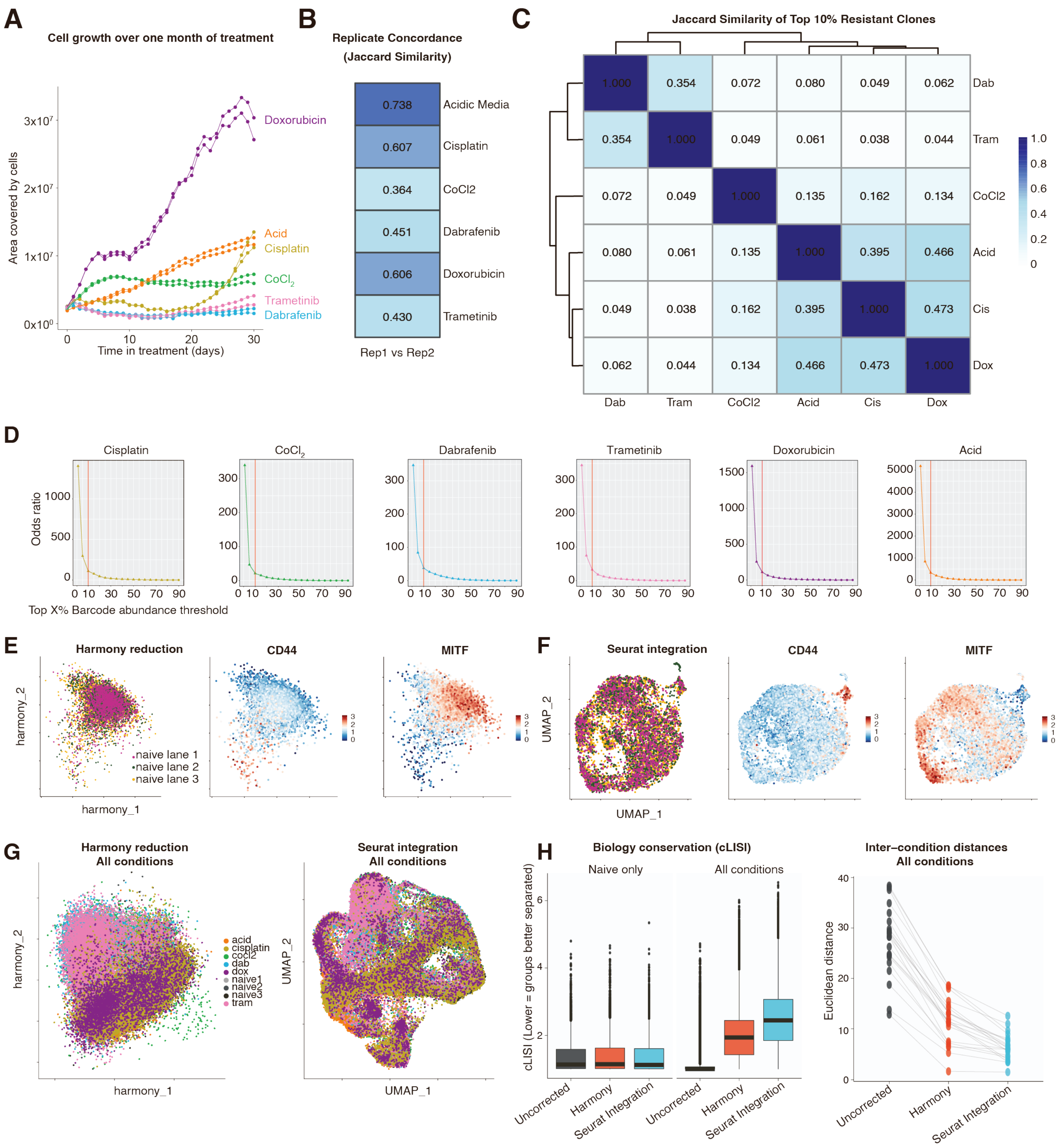

Figure S1: Cell growth, clonal concordance, and batch correction analysis, Related to Figures 1, 2, and 3.

A) Area covered by cells (in pixels) daily over the course of one month of treatment. Data is also shown in Figure 1B with variable y-axes per condition. **B)** Jaccard similarity for the top 10% of clones in each replicate of the same condition for the experiment shown in Figure 2A with data shown in Figure 2C. **C)** Pairwise Jaccard similarity indices across different combinations of treatment conditions. **D)** Sensitivity analysis of the top clone abundance threshold used for identifying resistant clones. Odds ratios for pairwise treatment comparisons are shown across varying thresholds (top 1% to 90%), demonstrating that enrichment of shared resistance is strongest at the most stringent cutoffs. **E)** Harmony batch correction and dimensionality reduction of naive scRNA-seq data. After batch correction, the expression of cell state marker genes CD44, MITF, and NT5E remain distinct, confirming that observed clusters represent biological heterogeneity rather than batch effects. **F)** Seurat integration for batch correction applied to the same data as in E. **G)** Harmony and Seurat integration applied to all treatment conditions, showing that batch correction merges biologically distinct treatment groups. **H)** Quantitative batch correction metrics for all conditions. Left: biology conservation (cLISI) for untreated cells and all conditions shows that correction across conditions increases cLISI, indicating loss of biological group separation. Right: inter-condition centroid distances in PC space collapse after correction, confirming overcorrection.

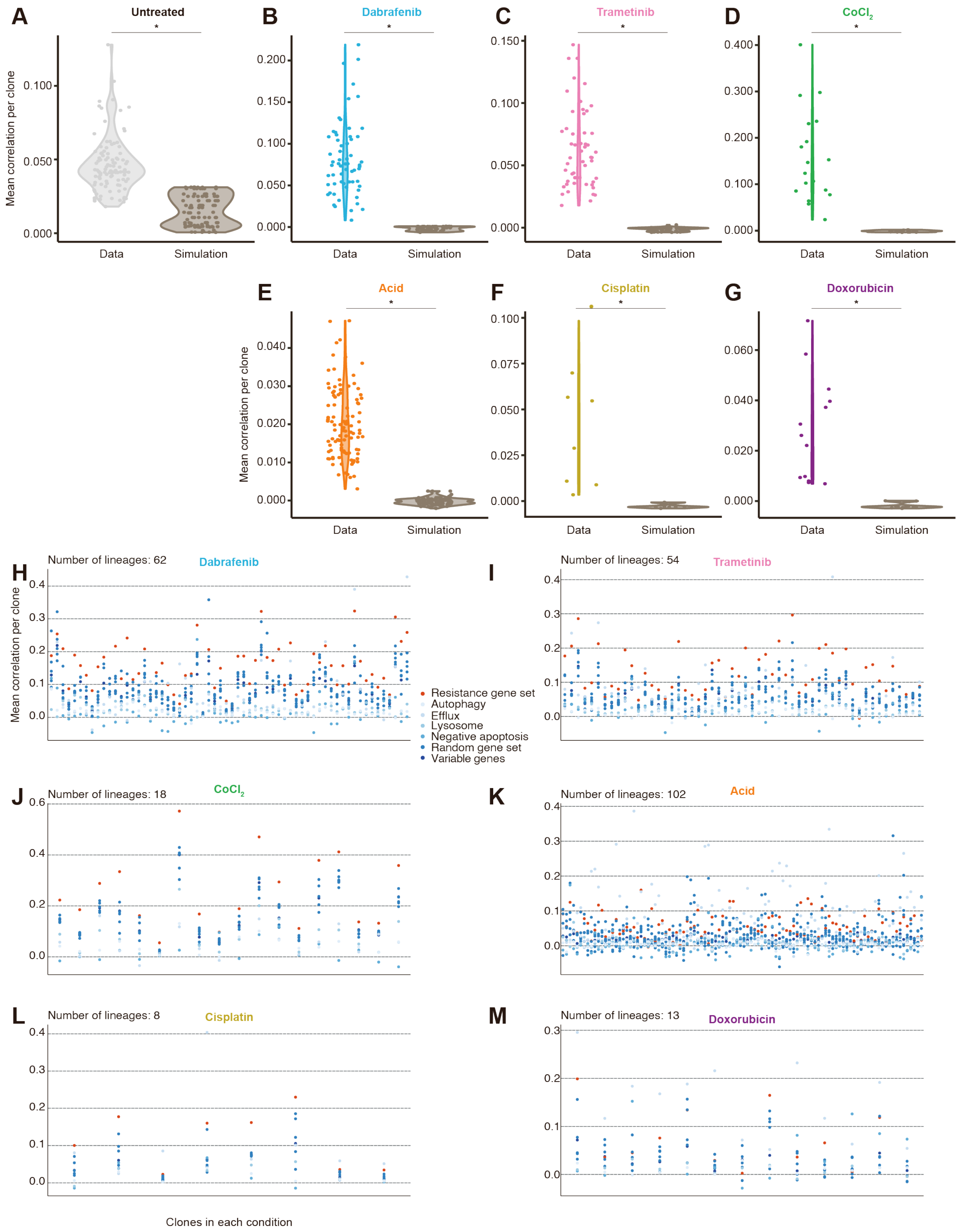

Figure S2: Cells within a clone are more transcriptionally similar than random samplings, with resistance-predictive genes showing the highest clonal coherence, Related to Figure 3.

A-**G)** Violin plots showing average pairwise Pearson correlations of all cells in a clone over the expression of the top 2000 most variable genes (left, colored) compared to size-matched simulated random clones (right, gray). For **A)** untreated clones, **B)** dabrafenib resistant clones, **C)** trametinib resistant clones, **D)** $CoCl_2$ resistant clones, **E)** clones resistant to acidic media, **F)** cisplatin resistant clones, and **G)** doxorubicin resistant clones, pairwise Pearson correlations among real clones were significantly higher than simulated clones in all 100 simulations by a one-sided Wilcoxon rank sum test with $p < 0.05$. H-**M)** Dot plots comparing intra-clonal correlations for resistance-predictive genes (red), random gene subsets matched for gene set size, the top 2000 most variable genes, and other functional gene sets (autophagy, efflux, lysosome, and negative regulators of apoptosis). Each plot shows the clones for a different treatment. Each dot represents the average pairwise correlation within a clone (minimum 15 cells). Resistance-predictive genes consistently show higher correlations than both random gene sets and variable genes, demonstrating clonal coherence for functionally relevant resistance mechanisms.

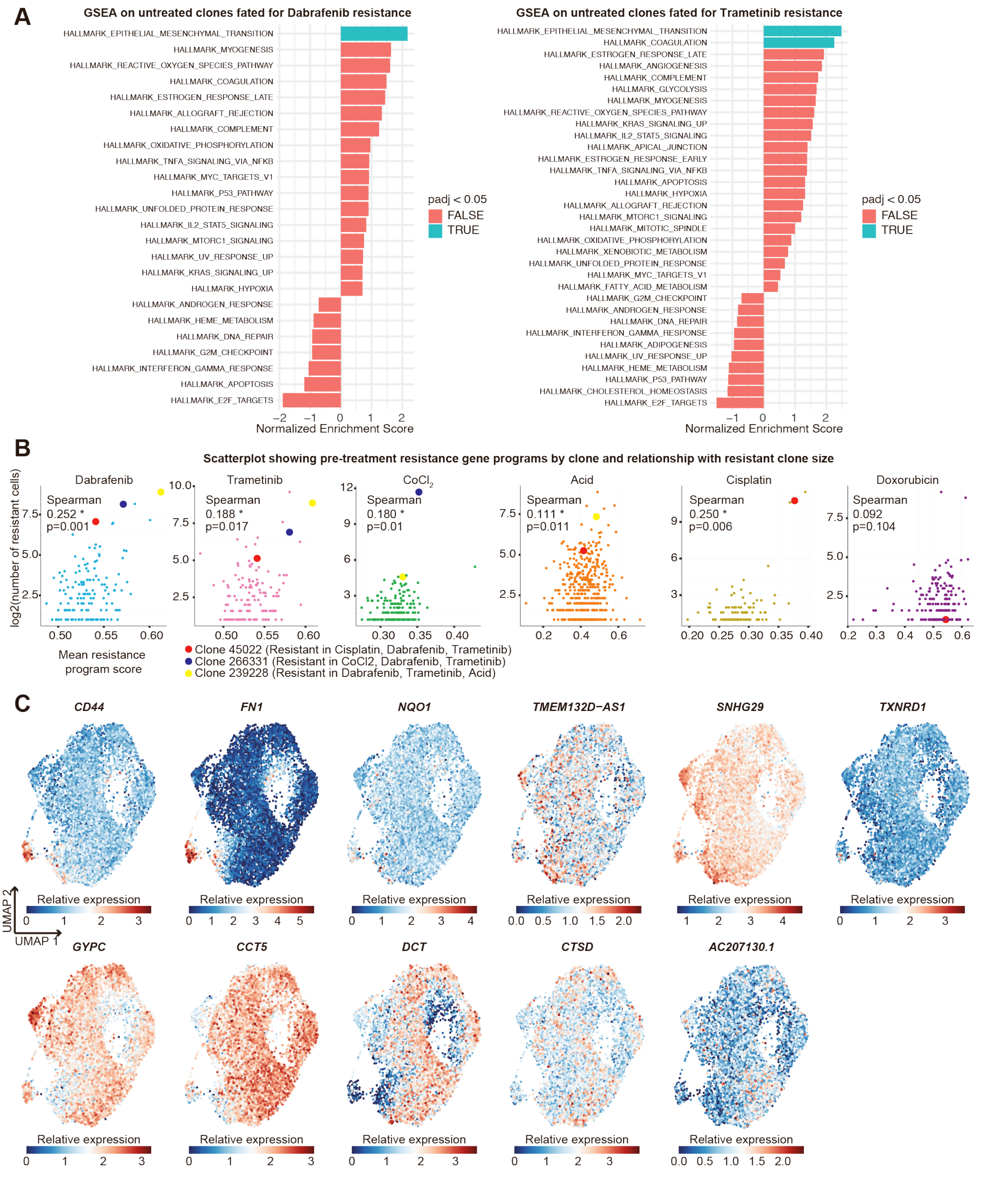

Figure S3: Gene set enrichment analysis, resistance program validation, and expression of multi-treatment resistance markers, Related to Figures 3 and 4.

A) Horizontal bar plots showing normalized enrichment scores of hallmark gene sets for genes upregulated in untreated clones that will develop resistance to dabrafenib (left) and trametinib (right). Significantly enriched gene sets (padj < 0.05) are colored teal. Both resistance signatures show enrichment for epithelial-mesenchymal transition, consistent with known mechanisms of BRAFi/MEKi resistance in melanoma. **B)** Scatter plots showing the relationship between mean pre-treatment resistance program scores (x-axis) and final resistant clone size (y-axis, $\log_2$(number of resistant cells)) for each treatment condition. Each point represents an individual clone that survived the given treatment. Spearman correlation coefficients are shown for each treatment with p-values. Three multi-treatment resistant clones from Figure 3E are highlighted in colored dots, demonstrating that these clones show elevated resistance program scores for the specific treatments to which they developed resistance. **C)** UMAP plots showing relative expression of 11 genes (CD44, FN1, NQO1, TMEM132D-AS1, SNHG29, TXNRD1, GYPC, CCT5, DCT, CTSD, and AC207130.1) that are markers associated with resistance to at least three different treatments in untreated cells. Expression levels are color-coded from low (blue) to high (red).

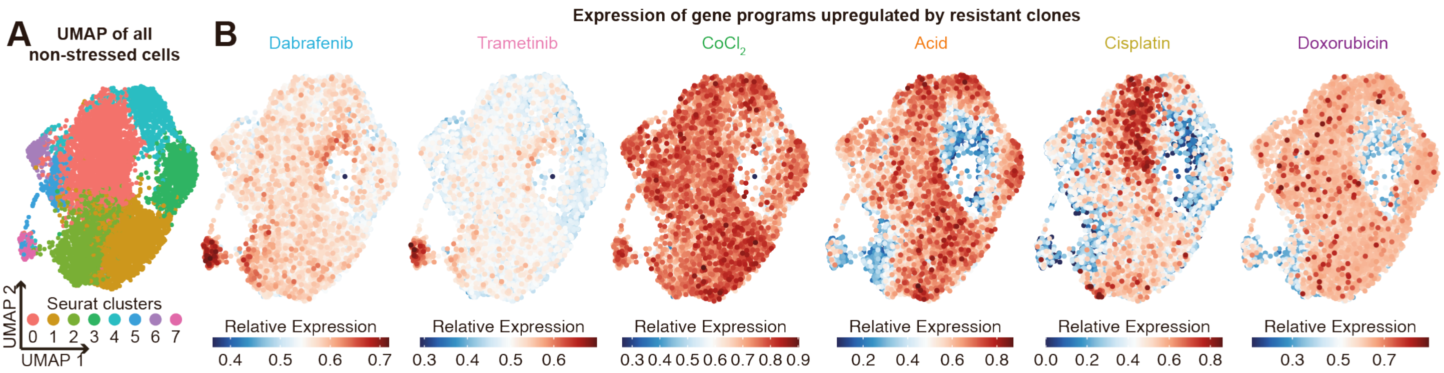

C

Marker genes upregulated in 3 or more conditions:
AC207130.1
CTSD
EEF1B2
GYPC
NQO1
RPS7
SNHG29

Figure S4: Gene expression signatures from the top 10 most resistant clones, Related to Figure 4.

A) UMAP of all untreated cells colored by Seurat clustering. **B)** UCell scoring of genes upregulated in the top 10 most resistant clones for each treatment condition, overlaid on the untreated cell UMAP. Expression levels are color-coded from low (blue) to high (red). Compared to the top five clone analysis (Figure 4B), dabrafenib and trametinib resistance signatures become more concentrated in the lower-left region, while $CoCl_2$, acid, and doxorubicin signatures show broader expression across multiple cell clusters. **C)** List of genes upregulated in at least three treatment conditions when analyzing the top 10 most resistant clones.

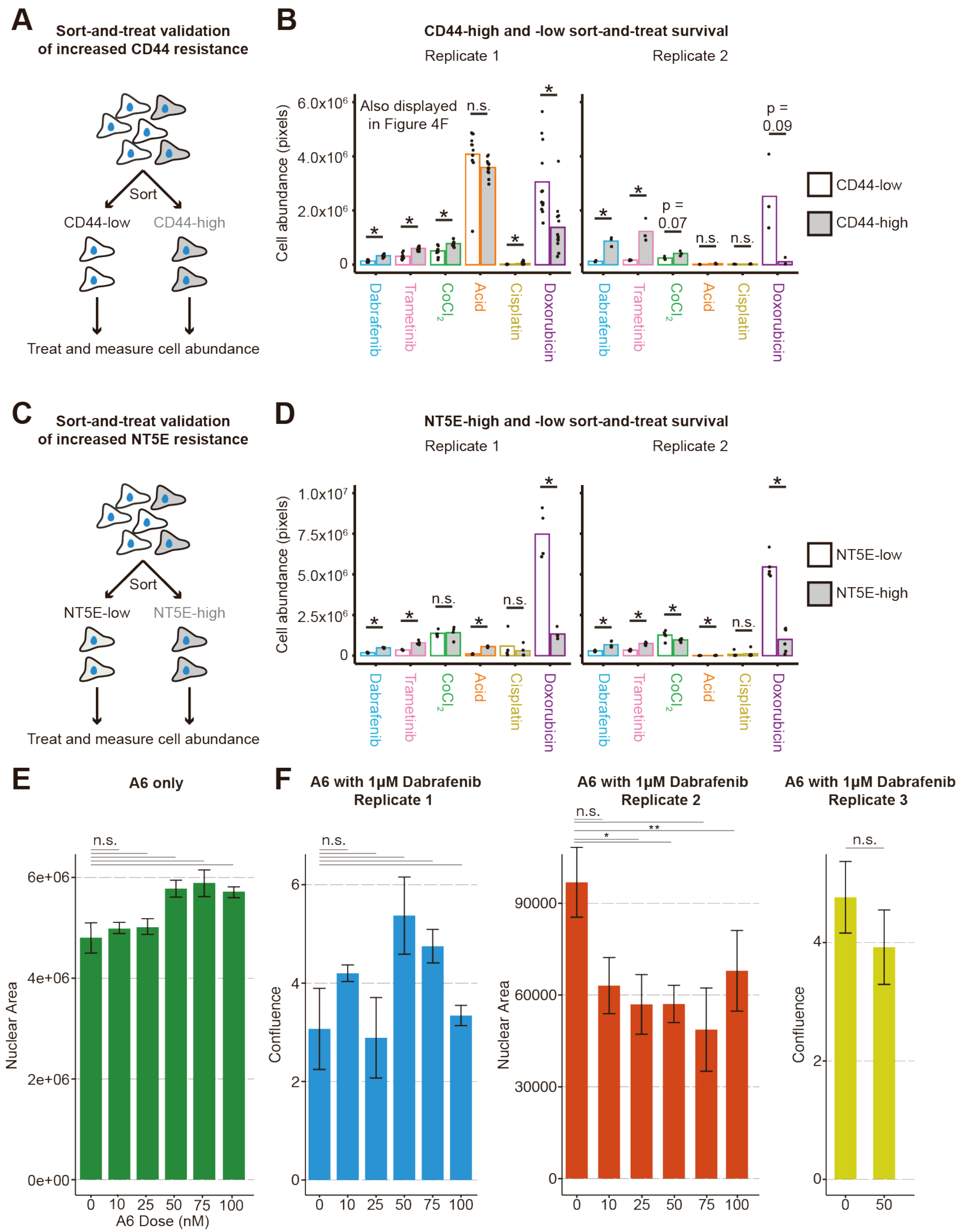

Figure S5: Assessing whether CD44-high or NT5E-high cells have multi-treatment resistance, Related to Figure 4.

A) Experimental protocol for sort-and-treat testing of whether CD44-high cells multi-treatment resistance. **B)** Results of CD44 sort-and-treat testing. Data is displayed as normalized cell abundance (in pixels) across two biological replicates. * indicates $p < 0.05$ by a Welch two sample t-test. **C)** Experimental protocol for sort-and-treat testing of whether NT5E-high cells multi-treatment resistance. **D)** Results of NT5E sort-and-treat testing. Data is displayed as normalized cell abundance (in pixels) across two biological replicates. * indicates $p < 0.05$ by a Welch two sample t-test. **E)** Results of A6 (Angstrom6) CD44 inhibitor treatment alone across increasing doses to assess potential toxicity. Data shows nuclear area with error bars representing standard error. A6 treatment alone did not significantly reduce cell viability. **F)** Results of A6 treatment in combination with 1 μM dabrafenib across three biological replicates and increasing A6 doses. Data shows confluence (replicates 1 and 3) or nuclear area (replicate 2) with error bars representing standard error. A6 did not consistently sensitize cells to dabrafenib treatment.

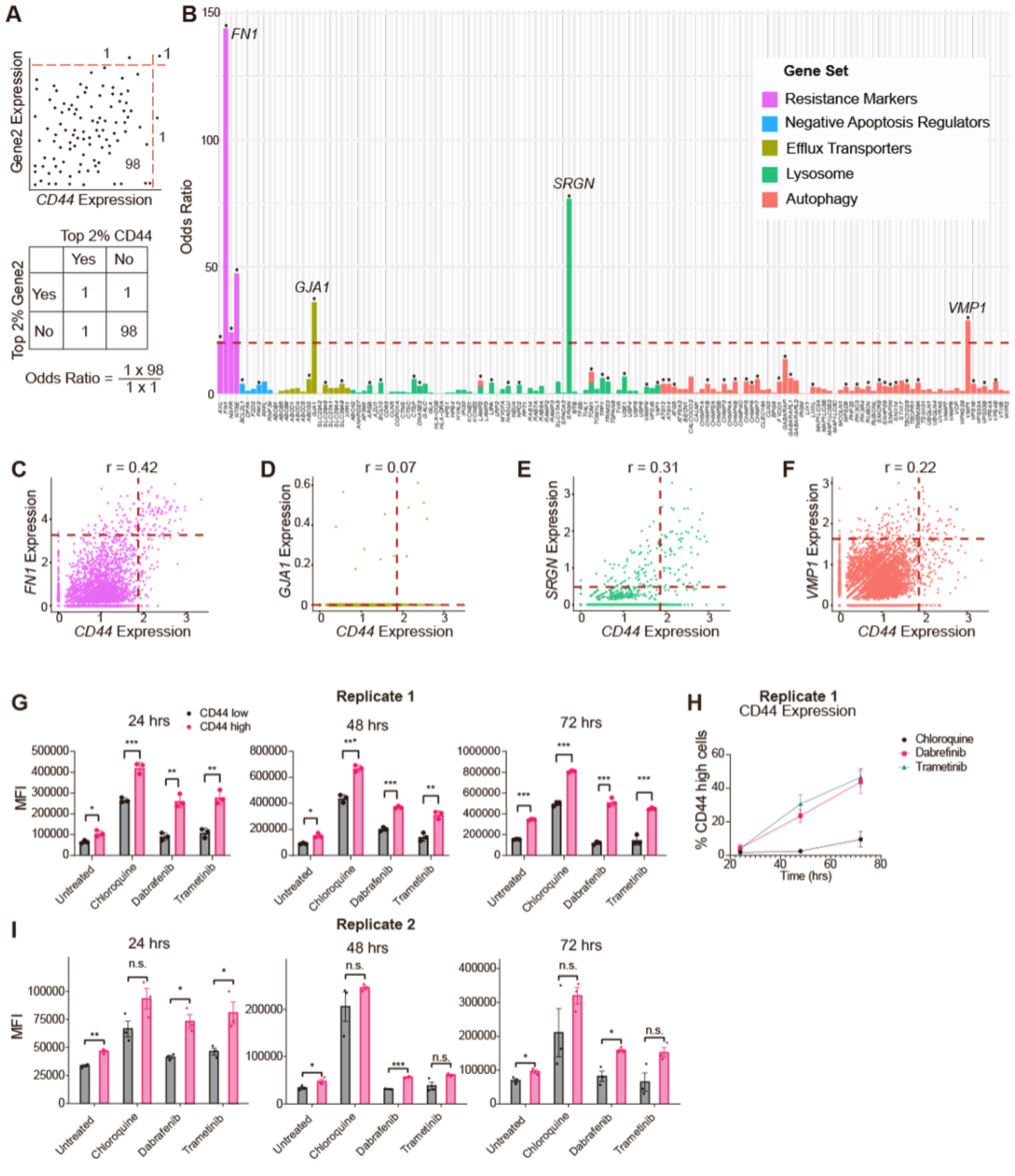

Figure S6: CD44-high cells show enrichment for lysosomal and autophagy pathway genes and elevated lysosomal activity, Related to Figure 4.

A) Representative analysis showing co-occurrence of CD44 and resistance pathway genes in the top 2% expressing cells, with contingency table demonstrating odds ratio calculation. **B)** Odds ratios for co-occurrence of CD44 with multidrug resistance pathway genes in the top 2% expressing cells. Genes are color-coded by functional category: known resistance markers (purple), negative apoptosis regulators (blue), efflux transporters (yellow), lysosome-related genes (green), and autophagy-related genes (red). Horizontal dashed line indicates odds ratio = 20. Notable enrichment is observed for lysosomal genes SRGN and VMP1. C-**F)** Scatterplots showing correlations between CD44 expression and key genes identified in B (FN1, GJA1, SRGN, VMP1) across all cells, with Pearson correlation coefficients indicated. G,**I)** Flow cytometry analysis of LysoTracker mean fluorescence intensity (MFI) in CD44-high (pink) versus CD44-low (black) cells across treatment conditions and time points in biological replicates 1 and 2. Treatments include untreated control, chloroquine (positive control), dabrafenib, and trametinib at 24, 48, and 72 hours. Statistical significance determined by t-test: * $p<0.05$, ** $p<0.01$, *** $p<0.001$; n.s., not significant. **H)** Time course analysis showing percentage of CD44-high cells over 72 hours across different treatment conditions.

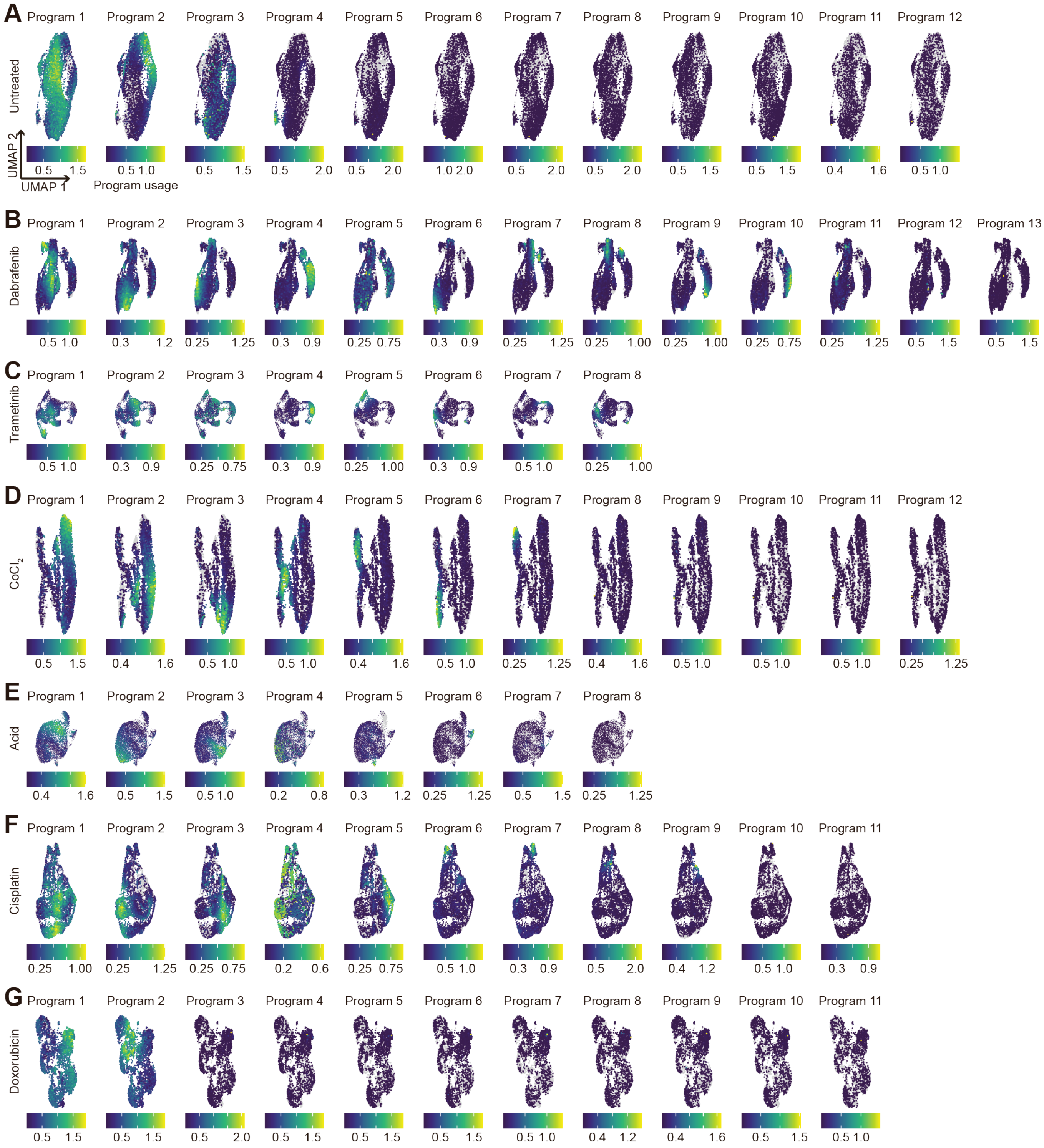

Figure S7: Cellular expression of cNMF gene programs for untreated and treatment-resistant cells, Related to Figure 5.

Cellular usage of cNMF programs for each condition. For every treatment condition, there were programs where we saw little to no variance in expression across cells, making them not informative. For each condition, the number of NMF programs with variable and informative expression are as follows: **A)** 4 programs for untreated cells, **B)** 11 programs for dabrafenib resistant cells, **C)** 8 programs for trametinib resistant cells, **D)** 7 programs for CoCl2 resistant cells, **E)** 7 programs for cells resistant to acidic media, **F)** 9 programs for cisplatin resistant clones, and **G)** 2 programs for doxorubicin resistant cells.

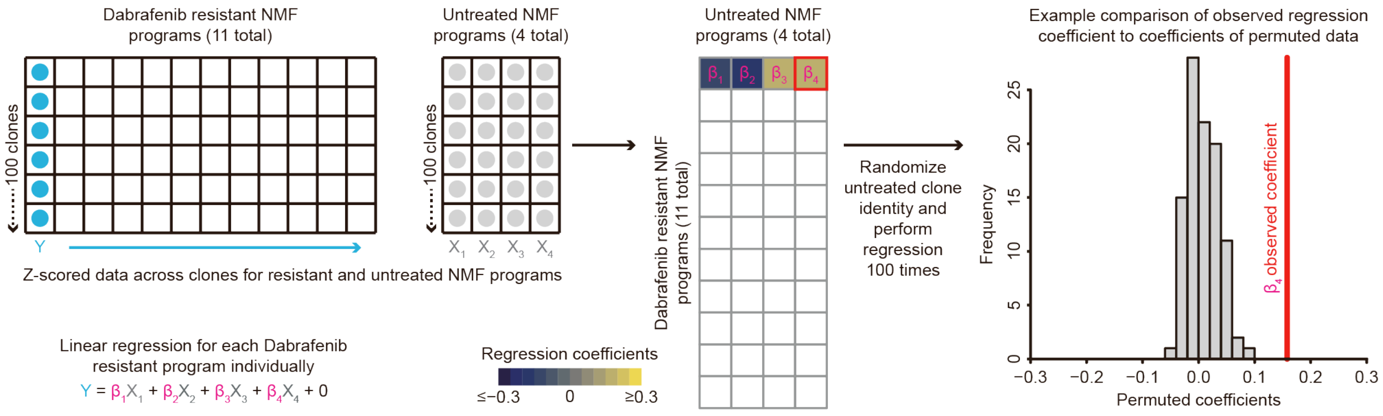

Figure S8: Schematic of mapping initial clonal gene expression state to end-state, Related to Figure 5.

We performed a linear regression of each resistant program individually against the four untreated NMF programs across the 100 most resistant clones for each condition. We then assessed the significance of these regressions by performing 100 simulations where we maintained the cellular identity of end-state clones but randomized the cells in each clone in the untreated population. We performed a Z-test to compare our observed regression coefficient against the permuted simulations.

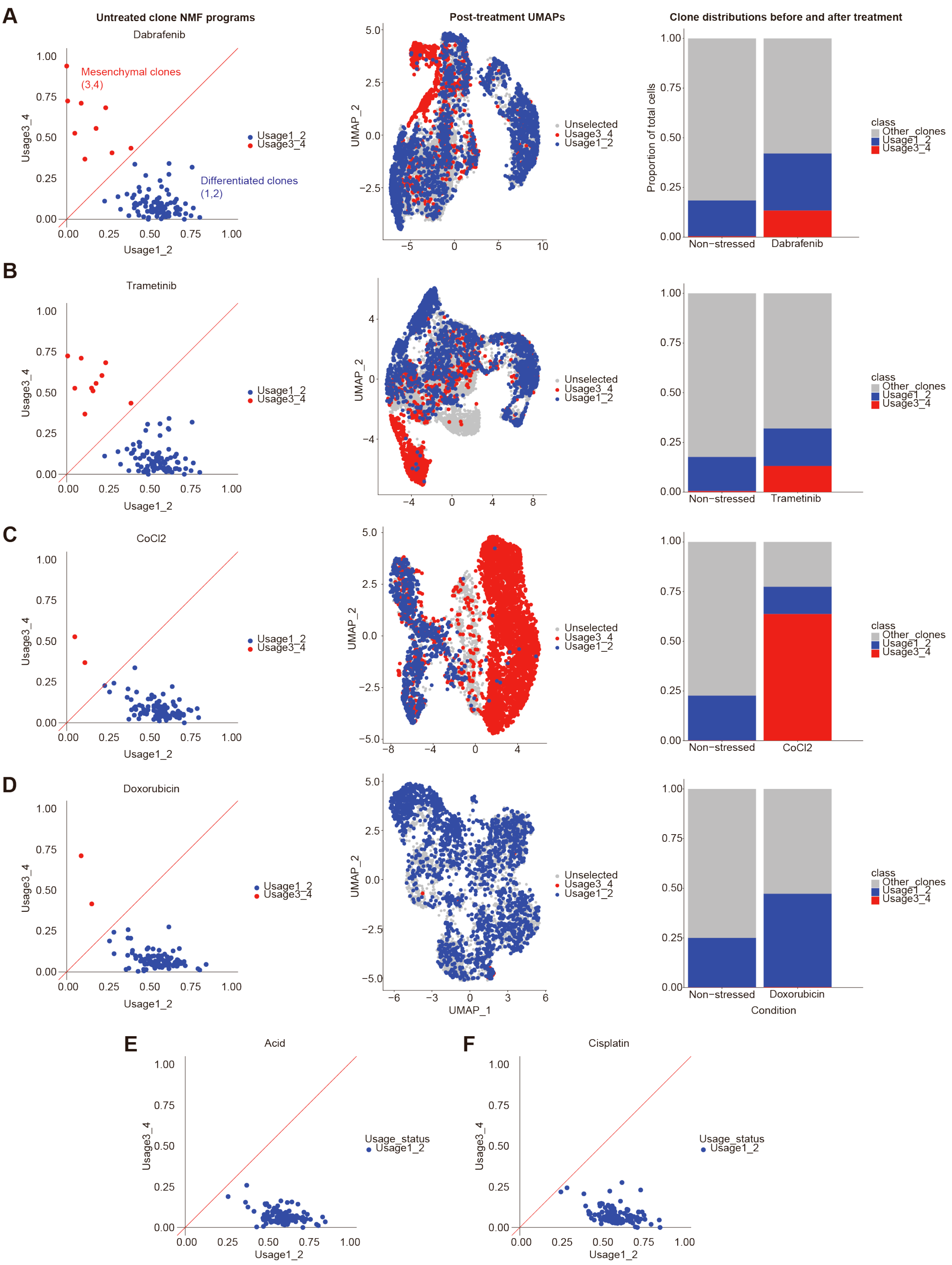

Figure S9: Initial clonal states predict distinct resistant outcomes across all treatment conditions, Related to Figure 5.

Analysis equivalent to Figure 5D-H extended to all treatment conditions. Left panels show scatter plots of clonal average expression of untreated NMF programs 1, 2 (differentiated, blue) versus programs 3, 4 (mesenchymal, red), with red diagonal line indicating equal expression (y=x). Middle panels show UMAP projections of resistant cells colored by their originating clonal classification, demonstrating spatial segregation of differentiated and mesenchymal-derived resistant cells. Right panels show stacked bar charts displaying the proportional representation of differentiated (blue), mesenchymal (red), and other clones (gray) before treatment (Non-stressed) and after resistance development. **A)** Dabrafenib (also shown in Figure 5), **B)** trametinib, **C)** $CoCl_2$, and **D)** doxorubicin show complete analysis with all three panel types. **E)** Acid and **F)** cisplatin only have scatter plots because the resistant cells are derived from clones that only express predominantly the untreated NMF usages 1 and 2.

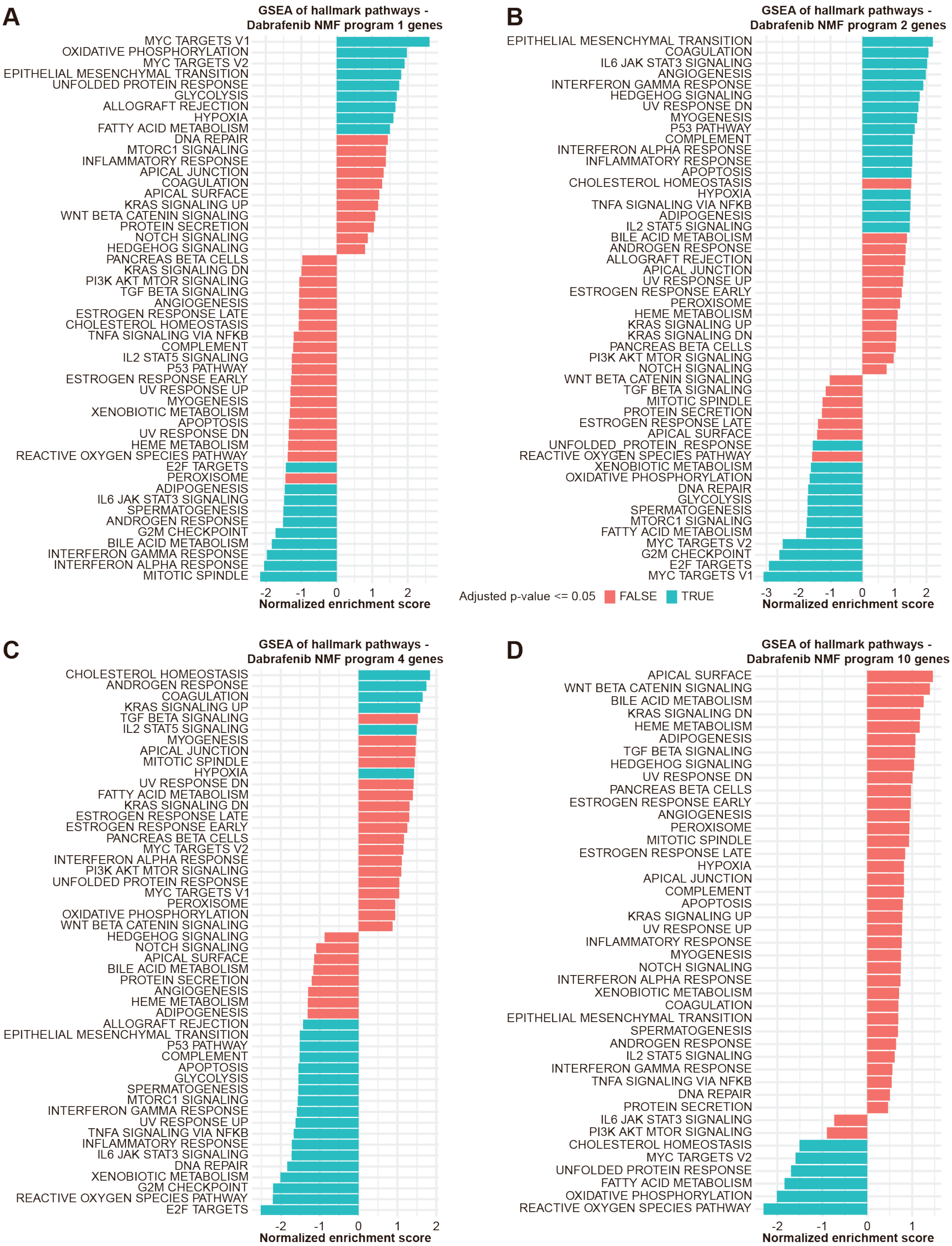

Figure S10: Gene set enrichment analysis of top genes in dabrafenib resistant NMF programs, Related to Figure 5.

Displayed are waterfall plots showing the normalized enrichment scores of GSEA [S1,S2] of hallmark gene sets [S3] for the top markers for the dabrafenib resistant NMF programs **A)** 1, **B)** 2, **C)** 4, and **D)** 10. Significantly enriched gene sets by adjusted p ≤ 0.05 are colored blue.

1. Korotkevich, G., Sukhov, V., Budin, N., Shpak, B., Artyomov, M.N., and Sergushichev, A. (2016). Fast gene set enrichment analysis. bioRxiv, 060012. https://doi.org/10.1101/060012.

2. Subramanian, A., Tamayo, P., Mootha, V.K., Mukherjee, S., Ebert, B.L., Gillette, M.A., Paulovich, A., Pomeroy, S.L., Golub, T.R., Lander, E.S., et al. (2005). Gene set enrichment analysis: a knowledge-based approach for interpreting genome-wide expression profiles. Proc. Natl. Acad. Sci. U. S. A. *102*, 15545–15550.

3. Liberzon, A., Birger, C., Thorvaldsdóttir, H., Ghandi, M., Mesirov, J.P., and Tamayo, P. (2015). The Molecular Signatures Database Hallmark Gene Set Collection. Cell Systems *1*, 417–425.
